# Reference Values of the QOLIBRI from General Population Samples in the United Kingdom and The Netherlands

**DOI:** 10.3390/jcm9072100

**Published:** 2020-07-03

**Authors:** Anastasia Gorbunova, Marina Zeldovich, Daphne C. Voormolen, Ugne Krenz, Suzanne Polinder, Juanita A. Haagsma, York Hagmayer, Amra Covic, Ruben G. L. Real, Thomas Asendorf, Nicole von Steinbuechel, on behalf of the CENTER-TBI Participants and Investigators

**Affiliations:** 1 Institute of Medical Psychology and Medical Sociology, University Medical Center Göttingen (UMG), Waldweg 37, 37073 Göttingen, Germany; Anastasia.Gorbunova@med.uni-goettingen.de (A.G.); marina.zeldovich@med.uni-goettingen.de (M.Z.); ugne.krenz@med.uni-goettingen.de (U.K.); amra.covic@med.uni-goettingen.de (A.C.); real.ruben@gmail.com (R.G.L.R.); thomas.asendorf@med.uni-goettingen.de (T.A.); 2 Department of Public Health, Erasmus MC, University Medical Center Rotterdam, P.O. Box 2040, 3000 CA Rotterdam, The Netherlands; d.voormolen@erasmusmc.nl (D.C.V.); s.polinder@erasmusmc.nl (S.P.); j.haagsma@erasmusmc.nl (J.A.H.); 3 Department of Emergency Medicine, Erasmus MC, University Medical Center Rotterdam, 3000 CA Rotterdam, The Netherlands; 4 Institute of Psychology, Georg-August-University, Goßlerstraße 14, 37073 Göttingen, Germany; york.hagmayer@bio.uni-goettingen.de

**Keywords:** QOLIBRI, disease-specific, health-related quality of life, traumatic brain injury, measurement invariance, reference values, healthy individuals, chronic health condition

## Abstract

The Quality of Life after Traumatic Brain Injury (QOLIBRI) instrument is an internationally validated patient-reported outcome measure for assessing disease-specific health-related quality of life (HRQoL) in individuals after traumatic brain injury (TBI). However, no reference values for general populations are available yet for use in clinical practice and research in the field of TBI. The aim of the present study was, therefore, to establish these reference values for the United Kingdom (UK) and the Netherlands (NL). For this purpose, an online survey with a reworded version of the QOLIBRI for general populations was used to collect data on 4403 individuals in the UK and 3399 in the NL. This QOLIBRI version was validated by inspecting descriptive statistics, psychometric criteria, and comparability of the translations to the original version. In particular, measurement invariance (MI) was tested to examine whether the items of the instrument were understood in the same way by different individuals in the general population samples and in the TBI sample across the two countries, which is necessary in order to establish reference values. In the general population samples, the reworded QOLIBRI displayed good psychometric properties, including MI across countries and in the non-TBI and TBI samples. Therefore, differences in the QOLIBRI scores can be attributed to real differences in HRQoL. Individuals with and without a chronic health condition did differ significantly, with the latter reporting lower HRQoL. In conclusion, we provided reference values for healthy individuals and individuals with at least one chronic condition from general population samples in the UK and the NL. These can be used in the interpretation of disease-specific HRQoL assessments after TBI applying the QOLIBRI on the individual level in clinical as well as research contexts.

## 1. Introduction

Traumatic brain injury (TBI) is often a source of long-lasting impairments and functional limitations [1]. It can affect participation in daily activities [2] and may lead to a stagnation in working life for several years [3] or permanently prevent a return to work [4]. TBI can have dramatic consequences for cognitive, behavioral, and emotional life domains, and increases the risk of experiencing other health-related problems such as increased alcohol consumption and depression [5]. However, a person’s perception of TBI sequelae, compared to an objectively assessed functional state, is a subjective dimension, and the relationship between these two types of measurement is not always straightforward [6]. Subjective assessments of health deficits and self-rated health-related quality of life (HRQoL) provide valuable additional information to clinical health examinations and ratings. Thus, patient-reported outcomes (PROs) have now become widely used in assessing HRQoL in the field of TBI. HRQoL measures provide aggregated information on diverse health components, such as physical, psychological (mental and emotional), social and daily life aspects, and are, therefore, able to capture the multidimensionality of individually experienced consequences of TBI [7].

A systematic review of assessments of HRQoL after TBI, covering the period from 1991 to 2013, found that the most frequently used instruments were the generic Short Form (36) Health Survey (SF-36) [8] and the TBI-specific Quality of Life after Traumatic Brain Injury (QOLIBRI) [1]. Both instruments display satisfactory to very good psychometric properties in TBI populations, with the QOLIBRI having higher discriminative powers when separate domains of the QOLIBRI and SF-36 are compared [7,9].

To gain a more in-depth understanding of TBI-specific consequences, one may apply a TBI-specific HRQoL instrument. However, from the perspective of rehabilitation after TBI, applying generic instruments may offer an advantage due to the availability of population-based reference values. Bearing in mind the unspecific nature of some post-TBI symptoms, such as headaches and nausea [10], a comparison with general population samples is essential in order to evaluate the rehabilitation progress. Additionally, population-based reference values play a key role in differentiating between individuals after TBI with and without impaired HRQoL.

In previous research, the QOLIBRI was developed and validated exclusively in samples of individuals after TBI to establish its sensitivity for the TBI condition [11]. In the interest of enhancing the interpretability of its scores in clinical practice and research after TBI, we collected QOLIBRI scores from general population samples in the UK and the NL to provide respective reference values.

Thus, the aims of the present study are:

- To ensure the comparability of QOLIBRI translations between general and TBI samples by determining the measurement invariance (MI) in general population samples (healthy individuals and individuals with a chronic health condition) and TBI samples from the UK and the NL.
- To provide reference values for healthy individuals and individuals with at least one chronic health condition from the UK and the NL.

Only when MI has been verified, reference values will be provided for healthy individuals (and individuals with a chronic health condition) from Dutch and UK general population samples. Separate reference values will be given for the presence and absence of chronic health conditions, age, sex, and level of education.

## 2. Methods

### 2.1. Study Design

The present study is a web-based, self-reported, cross-sectional study based on quota sampling of general population samples from the UK and the NL (see below). Additional data of patients after TBI, needed for the MI analyses, were retrieved from the multicenter, prospective, longitudinal, observational Collaborative European Neuro Trauma Effectiveness Research in Traumatic Brain Injury (CENTER-TBI) study [12]. These data were collected at three months post-TBI.

### 2.2. Setting

#### 2.2.1. General Population Samples

##### Data Collection

The general population sample data were collected through a web-based survey. Respondents were recruited by a market research agency (https://www.dynata.com/), which distributed the questionnaires and collected the data. The samples were based on existing large internet panels designed to be representative for individuals from the general population from the UK and the NL with regard to age, sex, and education. Data collection was carried out between 29 June and 31 July 2017.

The recruitment integrated several sources, e.g., proprietary loyalty partnerships (members of loyalty programs across travel, entertainment, retail, and other sectors), open recruitment to traditional online panels (e.g., via online banners, online all panels, cable TV advertising, mailings, social media influencers, and other methods), and integrated partnerships with online communities, publishers, and social networks. A broad variety of sources was chosen to reach participants from different social milieus to thereby increase the representativity of the sample.

To avoid a self-selection bias, no specific project details were included in the invitation: participants were invited to “take a survey”. Details were disclosed later, after the system had selected the individuals for participation according to the given selection criteria. After completing the survey, participants received an incentive in the form of cash, points, prizes, or sweepstakes from the market research company. Respondents, who were identified by the agency as “speeders” (e.g., who took the survey in less than five minutes), were deleted. The electronic data capture system did not allow missing answers, thus respondents had to answer every question. The recruitment process continued until the required quotas were reached.

##### Informed Consent

Informed consent for the present survey was obtained by the agency from all those agreeing to complete the online survey. The process is described in the privacy agreement available at https://www.dynata.com/privacy-policy/. Participants were informed on the welcome page of the survey that its aim was a better understanding of the consequences of TBI on patients’ lives, that it would take approximately 20 min to complete, and that all responses were confidential and anonymous. Data were anonymized and each participant was assigned a number in the order of questionnaire completion.

##### Sample Composition

From a total of 11,759 survey participants, 4646 individuals from the UK and 3564 from the NL were included for further analyses. Recruitment was carried out until the required quotas for age, gender, and education had been achieved, which ensured that samples were as comparable as possible to the general populations of the two countries. Nonresponse rates were below 20% (UK: 14.4%, NL: 19.5%). A more detailed analysis of these individuals was not possible due to the recruitment system used.

**Figure 1:**
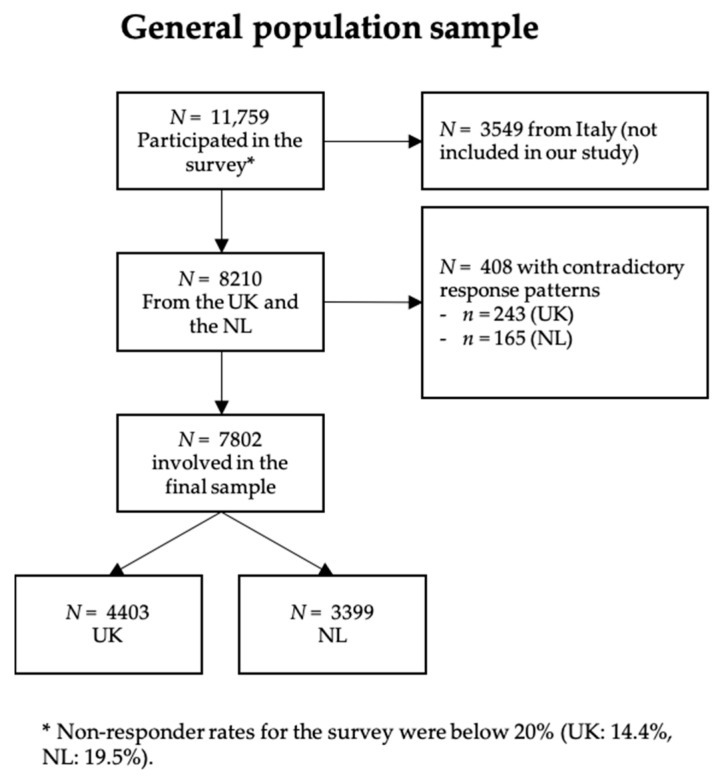
Sample attrition chart (general population).

Prior to the analyses, responses to QOLIBRI items were examined for obvious contradictory response patterns in both general population samples, for example, the choice of the response option “not at all” for all items, meaning that responders were not at all satisfied and at the same time not at all bothered. This indicated that the person had chosen only left-hand side response options, ignoring the item polarity. Due to contradictory response patterns, the data of 243 individuals from the UK and of 165 individuals from the NL general population samples were excluded from further analyses. The individuals included and excluded were compared using chi-square (χ^2^-) tests with Yates correction for nominal variables and independent *t*-test for continuous variables. In both countries, excluded individuals were predominantly male and younger compared with the total sample (*M* = 35, *SD* = 12) and had a middle level of education. In the end, 7802 individuals from the general population (UK: 4403; NL: 3399) were included in the final analyses (see Figure 1).

#### 2.2.2. TBI sample

##### Data Collection

Individuals after TBI were investigated in the (CENTER-TBI) study [13]. They were recruited between 9 December 2014 and 17 December 2017. The inclusion criteria were a clinical diagnosis of TBI, presentation to hospital within 24 h after the injury, a clinical indication for a computed tomography (CT) scan, and provision of informed consent adhering to local and national requirements. Data were collected applying an electronic case report form (e-CRF, QuesGen Systems Incorporated, Burlingame, CA, USA) either during the hospital visit, in a face-to-face visit, a telephone interview, or by mail combined with a telephone interview. The data were exported from the CENTER-TBI database, Neurobot version 2.0, on 8 November 2018. Further study details can be found elsewhere [12].

##### Informed Consent

Informed consent was obtained according to local and national requirements for all patients recruited in the Core Dataset of CENTER-TBI and documented in the e-CRF [13].

##### Sample Composition

Out of the total of 4509 CENTER-TBI core study participants, 554 individuals after TBI from the UK and 936 from the NL participated in the assessments at three months post-TBI and were included in the present study. When there were less than 30% of missing answers per QOLIBRI subscale, scores were calculated by using the prorating method [14]. Of the 1490, 830 individuals did not complete the QOLIBRI at three months.

**Figure 2:**
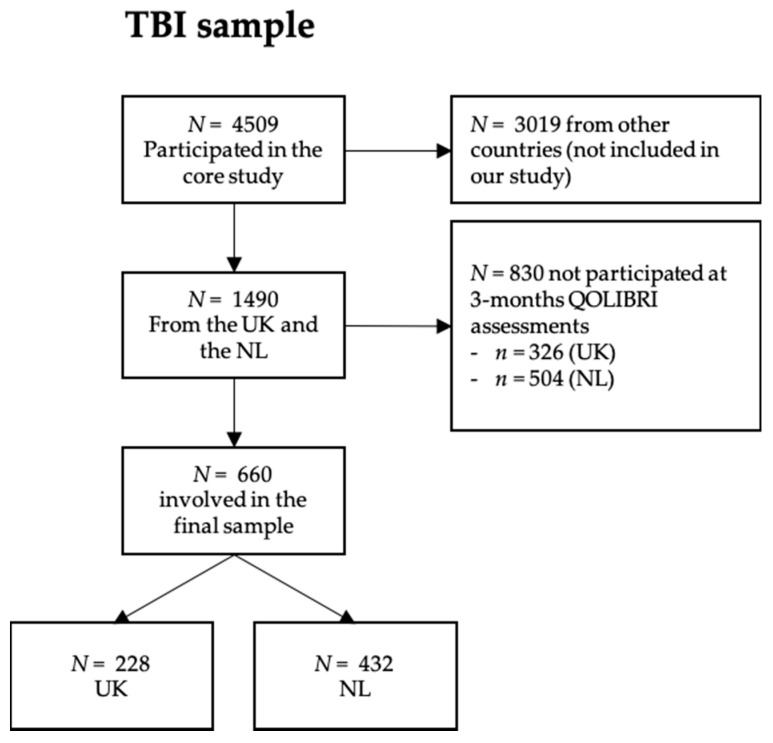
Sample attrition chart (traumatic brain injury (TBI) sample).

Chi-square tests with Yates correction for nominal variables and independent *t*-test for continuous variables showed that participants from the NL had a higher level of education, were mostly female, working or studying, and had predominantly sustained a mild TBI (84% in the NL and 72% in the UK) with a good recovery rated by the Glasgow Coma Scale Extended (GOSE) [15], compared to those who did not complete the QOLIBRI. Analyses of contradictory response patterns did not reveal any peculiarities. No exclusion based on QOLIBRI response patterns was necessary for the TBI sample. A total of 660 individuals (UK: 228, NL: 432) were, therefore, included in the further analyses. For more details on TBI sample attrition, see Figure 2.

### 2.3. Ethical Approvals

#### 2.3.1. General Population Sample

The study on the general population sample was part of the CENTER-TBI study and ethical approval was obtained from the Leids Universitair Centrum—Commissie Medische Ethiek (approval P14.222/NV/nv).

#### 2.3.2. TBI Sample

The CENTER-TBI study (EC grant 602150) was conducted in accordance with all relevant laws of the European Union, which were directly applicable or had a direct effect, and all relevant laws of the countries in which the recruiting sites were located, including but not limited to, the relevant privacy and data protection laws and regulations (the “Privacy Law”), the relevant laws and regulations on the use of human materials, and all relevant guidance relating to clinical studies including, but not limited to, the ICH Harmonised Tripartite Guideline for Good Clinical Practice (CPMP/ICH/135/95, “ICH GCP”) and the World Medical Association Declaration of Helsinki entitled “Ethical Principles for Medical Research Involving Human Subjects”. Ethical approval was obtained for each recruiting site. The list of sites, ethical committees, approval numbers, and approval dates can be found on the project’s website https://www.center-tbi.eu/project/ethical-approval.

### 2.4. Sociodemographic and Health Status Data of the All Samples

All study participants provided information regarding their age, sex, and level of education. Individuals from the general population samples were asked if they had one or more chronic health conditions (asthma, heart disease, stroke, diabetes, back complaints, arthrosis, rheumatism, cancer, memory problems due to a neurological condition like dementia, memory problems due to aging, depression, or other problems). Multiple answers were allowed.

The severity of TBI was rated by attending clinical personnel using the Glasgow Coma Scale (GCS), with values of 3–8 indicating severe, 9–12 moderate, and 13–15 mild TBI [16]. Recovery after TBI was rated using the Glasgow Outcome Scale Extended (GOSE) with scores of 3–4 indicating severe, 5–6 moderate disability, and 7–8 good recovery. Scores of 2 indicate a vegetative state and a score of 1 death [15].

#### Disease-Specific Health-Related Quality of Life after Brain Injury (QOLIBRI)

HRQoL was assessed administering the TBI-specific QOLIBRI questionnaire, which was developed and validated in accordance with the World Health Organization definition of health [14,17]. It covers six life domains (Cognition, Self, Autonomy and Daily life, Social Relationships, Emotions and Physical Problems). Items contributing to the domains Emotions and Physical problems are negatively worded (“How bothered are you by…?”), the remaining items positively (“How satisfied are you with…?”). Thirty-seven items are rated on a 5-point Likert scale (“Not at all” = 1, “Slightly” = 2, “Moderately” = 3, “Quite” = 4, “Very” = 5) and reverse coding was performed for negatively worded items. The QOLIBRI total score is scaled to vary between 0 (worst possible HRQoL) and 100 (best possible HRQoL) [14].

As not all items were directly applicable to the general population, three items were reworded to remove any reference to a TBI: “How satisfied are you with what you have achieved recently (instead of “since your brain injury”)?”, “How bothered are you by the effects of any injuries you sustained? (instead of “any other injuries you sustained at the same time as your brain injury”)”, and “Overall, how bothered are you by the effects of any health problems? (instead of “brain injury”)”.

### 2.5. Statistical Analyses

The statistical analyses comprised the following steps: (1) examination of the psychometric properties of the QOLIBRI on the item and scale level in the general population; (2) MI analyses between groups of individuals from the TBI and general population samples and between the countries, to ensure that the same concept of HRQoL was being measured; (3) multivariate linear regression analyses, which examined whether country of residence, age, sex, level of education, and the presence of chronic health conditions affected the HRQoL/QOLIBRI total score; (4) based on the regression results, computation of reference values for individuals with and without chronic health conditions for the QOLIBRI total score and subscales with respect to age, sex, and level of education.

Descriptive statistics (mean, standard deviation, response frequencies) were used to describe participants’ sociodemographic and health-related data.

#### 2.5.1. Item Characteristics of the QOLIBRI in the General Populations

As the main focus of this study was to provide reference values for the QOLIBRI from general population samples, item properties such as mean, standard deviation, skewness, and ceiling effects are only reported for the general population samples. Items with absolute skewness values between 1.0 and 1.3 were interpreted as moderately skewed and not affecting further analysis [10,18]. Due to the high variation in cut-off values for ceiling effects (15–60%) in the current literature [10,19], we set the cut-off value at 40% (twice as high as by chance, 1/5 = 20%) for the maximum response category “very”. Additionally, we checked if there were items with less than 10% of responses in the two lower response categories “not at all” and “slightly”.

#### 2.5.2. Scale Characteristics of the QOLIBRI in the General Populations

The scales’ internal consistency was determined using Cronbach’s alpha, with values between 0.7 and 0.95 indicating good to excellent internal consistency [19]. An item was defined as inconsistent when the corrected item-total correlation coefficient (CITC) exceeded 0.4 [20]. Correlations between the QOLIBRI domains were investigated using Pearson correlation coefficients, with values ranging from 0.36 to 0.67, indicating a moderate linear association [21].

#### 2.5.3. Construct Validity of the QOLIBRI in the General Populations

As a prerequisite for MI testing, construct validity was investigated in the general population samples to ensure the comparability of the reworded and the original QOLIBRI using confirmatory factor analysis (CFA) with the robust weighted least squares estimator (WLSMV, calculated with the lavaan-package in R [22]). Model fit was assessed by means of the scaled chi-square statistics, Comparative Fit Index (CFI), and root mean square error of approximation (RMSEA) with a 90-percent confidence interval. As the standard cut-offs for CFI (>0.95) and RMSEA (<0.06) [23,24], indicating good model fit, have not been validated for the WLSMV estimator, and they should be interpreted with caution [25]. To address this issue, we compared fit indices across models with different factorial structures (one common factor, two correlated factors—one containing all positively worded “satisfaction” items, and the other one all negatively worded “bothered” items, and six correlated factors) with higher CFI values and lower RMSEA values indicating a better model.

#### 2.5.4. Measurement Invariance in All Samples

By using modern statistical techniques, such as MI testing, it is possible to verify whether the questionnaire score differences between individuals, e.g., with and without TBI experience, can be attributed to true differences in HRQoL or rather to differences in interpretation of the items and response categories, as well as differences in items difficulty and their importance [26].

Therefore, MI testing in the framework of CFA was applied to examine whether TBI experience and cultural/language differences influenced the comprehension of the QOLIBRI items. First, we examined the influence of the TBI experience on the invariance of model parameters by comparing groups of individuals from the TBI and general population samples separately for each country. To overcome estimation problems due to the large number of estimated parameters and relatively small sizes of the two TBI samples, the QOLIBRI items were dichotomized. The response categories “not at all”, “slightly” and “moderately” were coded as 0, and “quite” and “very” as 1. We then investigated the effect of the country by comparing UK and NL general population samples.

The strategy for analyzing ordinary scaled response categories suggested by Wu and Estabrook (2016) was applied, resulting in three steps: testing of the (1) configural, (2) partial, and (3) full invariance model. For more details, see Wu and Easterbrook [27].

For MI analyses, at least *N* = 200 observations per group are necessary to obtain reliable results [28]. All estimations for invariance testing (WLSMV-estimator, theta-parameterization) were performed within the lavaan-package (version 0.6-3) [22]. For model comparisons, we applied a scaled chi-square difference test with the significance level set to α = 0.05. As this test has been criticized for being very powerful in detecting small, possibly irrelevant effects in large samples [29], in case of invariance violation, we estimated whether the effect had a practical significance for estimating the probability of choosing a particular response category. For example, if the full invariance model (invariant thresholds) had a significantly worse fit than the partial invariance model (noninvariant thresholds), the probabilities of individuals from general population samples choosing a particular response category were estimated in both models, and then compared. If the differences did not exceed 5%, we considered the thresholds to be invariant [30].

#### 2.5.5. Reference Values from General Population-Based Samples

As clinicians may be interested in the subjective health status and HRQoL of a single patient after TBI, population-based reference values were calculated as percentiles. Percentiles indicate the value below which a given percentage of observations falls. Based on this information, one can determine whether the QOLIBRI score of an individual after TBI is below, equal to, or above the value of the reference population. The following percentiles are provided for a patient-level interpretation: 2.5%, 5%, 16%, 30%, 40%, 50%, 60%, 70%, 85%, 95%, and 97.25%. HRQoL is considered to be impaired when scores are one standard deviation below the average of the general population sample [31], which corresponds to the 16%-quantile when the data are assumed to be normally distributed. Examples are given in the results section.

Previous research has shown that 50 to 75 cases for each subgroup can already be sufficient to provide norm values [32]. However, as several factors can influence the required sample size (e.g., which type of norms are provided [33]), we have decided to report reference values when the number of cases was at least *N* = 100. All analyses were performed in R 3.6.0 [34].

## 3. Results

### 3.1. Sociodemographic and Health-Related Data

#### 3.1.1. General Population Sample

Study participants (*N* = 4403 from the UK and *N* = 3399 from the NL) from the general population samples were analyzed. Individuals without a chronic health condition (UK: 2016; NL: 1572) were differentiated from individuals with chronic health conditions (UK: 2387; NL: 1827; for details, see Table 1). In both countries, up to 55% of individuals from the general population samples indicated that they had at least one chronic health condition, and, in comparison with the TBI samples, significantly more individuals described themselves as being unable to work (UK: 10%, NL: 12.8%).

#### 3.1.2. TBI Sample

The TBI sample contained 660 individuals (*N* = 228 from the UK and *N* = 432 from the NL), who had filled in the QOLIBRI at three months post-TBI. The majority of individuals from both TBI samples had experienced a mild TBI (71.9% and 84.1 % in the UK and NL, respectively). In the UK, almost half of all individuals after TBI made a good recovery 48.7% (NL: 66.2%) and 20% were still severely disabled (NL: 8.8%) at three months post-TBI. Sociodemographic and health-related data for all samples are presented in Table 1.

#### 3.1.3. Comparison of the General Population Samples with TBI Samples

In both countries, significant differences were identified between the general population samples and the TBI samples concerning age, sex, educational level, and work status. In both general population samples, individuals were younger than in the TBI samples (with an average age difference of five years in the UK and of 10 years in the NL) and had a lower male incidence (UK: 48.5% vs. 66.7%, NL: 49.0% vs. 58.6%). The rate of individuals with a high level of education (diploma, secondary/high school, or post-high school) was lower in the general population samples compared with the TBI samples (UK: 34.5% vs. 43%; NL: 25% vs. 31.7). In the UK, the number of working individuals was lower in the general population sample compared with the TBI sample (51.5% vs. 63.6%, respectively), whereas in the NL the general population sample contained more employed individuals compared with the TBI sample (52.3% vs. 46.8%, respectively).

### 3.2. Item Characteristics of the QOLIBRI in the General Population Samples

On a descriptive level, there were some differences between countries concerning the item characteristics: individuals from the UK general population sample scored lower on average but with higher dispersion and mean values varying from 3.0 (satisfaction with sex life) to 4.1 (satisfaction with the ability to find a way around; NL: from 3.5 to 4.2); items were less skewed ((−1; −0.2), NL: (−1.3; −0.3)), and a ceiling effect was observed for only six items, compared with 10 in the NL sample. All items in the UK sample and 22 items in the NL sample had over 10% responses in two adjusted response categories “not at all” and “slightly”. For more detailed information, see Appendix A Table A1.

### 3.3. Scale Characteristics of the General Population Samples

The total scale Cronbach’s alpha was high in both general population samples (UK: 0.94, NL: 0.96), and per-scale alpha coefficients ranged between 0.86 (Emotions) and 0.95 (Cognition) in the UK general population sample, and between 0.86 and 0.92 in the NL, indicating a very good internal consistency of the scales. Also based on CITC, all items were found to be consistent in both samples. QOLIBRI domains were moderately to highly correlated (UK: 0.39–0.77, NL: 0.46–0.76). For more detailed information, see Appendix A Table A2.

### 3.4. Construct Validity of the General Population QOLIBRI

Based on CFA results, a six-factorial structure was most appropriate for the QOLIBRI in the UK (χ^2^(614) = 15,441, *p* < 0.001, CFI = 0.957, RMSEA = 0.074, 90%CI (0.073; 0.075)) and also in the NL (χ^2^(614) = 10,276, *p* < 0.001, CFI = 0.952, RMSEA = 0.068, 90%CI (0.067; 0.069)) general population samples. For more detailed information, see Appendix A Table A3.

### 3.5. Measurement Invariance

When the general population and TBI samples were compared for each country, the fit of the model with six correlated factors was not negatively affected by constraining equal intercepts, loadings, and residuals across all groups (UK: Δχ^2^(Δdf) = 23.00 (25), *p* = 0.577, NL: Δχ^2^(Δdf) = 8.27 (25), *p* = 0.999). However, assuming equality of thresholds resulted in significantly higher chi-square values, indicating that some thresholds may not be invariant across groups. When UK and NL general population samples were compared, significant, yet very small, and thus, negligible chi-square differences (Δχ^2^(Δdf) = 87.27 (25), *p* < 0.001) were observed [32]. The model fit deteriorated meaningfully when the thresholds were restricted to be equivalent across groups (Δχ^2^(Δdf) = 2395.26 (148), *p* < 0.001). For details, see Appendix A Table A4.

**Figure A1:**
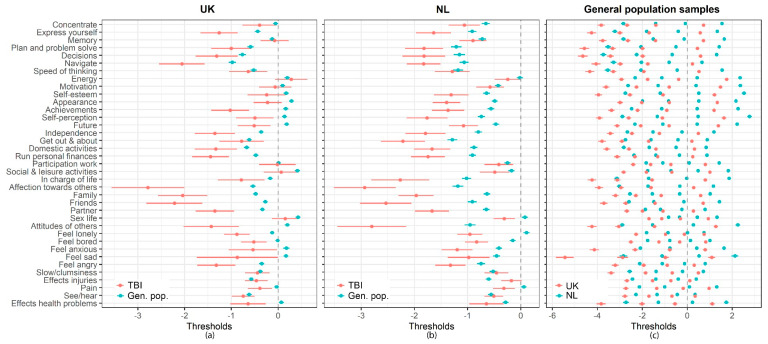
(**a**) Comparison of the thresholds estimated with the partial invariance model for the UK TBI and general population samples; (**b**) Comparison of the thresholds estimated with the partial invariance model for the NL TBI and general population samples; (**c**) Comparison of the thresholds estimated with partial invariance model for the UK and NL general population samples.

More detailed analyses on the estimated thresholds using the partial invariance model showed that the thresholds obtained in the general population sample were significantly higher than those in the TBI sample. Comparing the UK and NL general population samples, the thresholds obtained from the UK sample were lower in all cases (see Appendix A, Figure A1). However, for individuals from the general population samples, differences in the probabilities of choosing a particular response category did not exceed 5% (Appendix A, Table A5). Therefore, the violation of the threshold invariance may be interpreted as not significant. This implies that the QOLBRI scores can be compared between countries, and between the general population and TBI samples. More important, differences in the QOLIBRI scores should be attributed to “real” differences in HRQoL.

### 3.6. Reference Values for the General Population Samples

A significant difference in HRQoL as indicated by the QOLIBRI total score, was found between the countries. The NL sample experienced a significantly higher HRQoL compared with the UK general population sample (β = 8.76, *p* < 0.001). Regression analyses identified a significant effect of age, level of education, presence of at least one chronic health condition, and interactions between age and sex and health status in both general population samples. No significant effects for sex were found in both general population samples (Table 2).

Reference values of the general population-based samples for the QOLIBRI total score are presented in Table 3 for the UK and Table 4 for the NL. The tables with the reference values for the QOLIBRI subscales can be found in the Online Supplementary Materials (Table S1: UK; Table S2: NL).

The following example illustrates how to apply these norms. After a TBI, a 70-year-old woman from the UK without any chronic health condition reports a QOLIBRI total score of 75. The table depicts that around 20% of healthy individuals in her age group reported the same level of HRQoL or a lower HRQoL. In other words, 80% of the reference population experience better HRQoL. Should a chronic health condition be known, 60% of the reference population from her age and health status group report better HRQoL and 40% of the general population with similar conditions experience a better HRQoL than she does.

Based on the 16%-percentile cut-off value, HRQoL is interpreted as impaired for female healthy individuals in the age range of 64–75 years when the QOLIBRI total score is under 69, or under 50 if any chronic health condition is reported. The score of 75 exceeds both cut-off values and can, therefore, be interpreted as indicating that she is not impaired (compared with individuals from the UK general population aged between 65–75 years with and without any chronic health condition).

## 4. Discussion

The aim of our study was to enhance the interpretability of disease-specific HRQoL after TBI using the QOLIBRI by establishing reference values from general population samples in the UK and the NL, based on representative quotas with regard to sex, age, and educational level. The representation of these characteristics corresponds to their distribution in the UK and the NL general populations (e.g., see the Organisation for Economic Co-operation and Development (OECD) [35] for sociodemographic characteristics in European countries). In this respect, the data from our general population samples are comparable to the general population of each country. This study is unique, as such general population-based reference values are currently not available for the QOLIBRI.

The results indicated that the reworded QOLIBRI is applicable to general population samples and displays good psychometric properties. Measurement invariance testing demonstrated that for the six HRQoL subdomains, all QOLIBRI items have the same meaning for individuals with and without a TBI experience and in the different countries. Therefore, we conclude that the QOLIBRI scores can be compared across general population samples and TBI samples in the UK and the NL. The differences in the scores have to be explained by “real” differences in HRQoL and not by other factors, such as differences in the understanding of items or response categories. Thus, we were able to establish population-based reference values.

In previous research, individuals from the NL general population reported higher mental summary component scores in the SF-36 in comparison to seven other countries, including five European countries [36]. Lower HRQoL was associated with the presence of chronic health conditions [37]. Our results replicate these findings, with individuals from the NL general population sample reporting significantly higher HRQoL compared to those from the UK. Previous findings concerning the association of HRQoL with age are ambiguous: in the general populations of Norway and Canada, higher age was positively associated with the mental summary component score of the SF-36 and negatively with the physical summary component [38,39]. Our data showed that older individuals from the general population samples from both countries and subsamples with and without chronic health conditions report better HRQoL. Our study did not identify any sex differences in the two countries, with the exception of the subgroups with and without any chronic health conditions in the NL sample. This finding is also comparable to a study assessing the generic HRQoL by means of the SF-36: here only the general health perception scale was sensitive to sex differences, with females reporting lower generic HRQoL [8,40].

Previously, the interpretation of the QOLIBRI total score was facilitated through a cross-walk analysis with the mental component summary score of the SF-36, for which US population-based norms were used [41]. HRQoL was considered to be impaired when scores were one standard deviation below the average of the general population sample [35]. Therefore, QOLIBRI values under 60 indicated impaired disease-specific HRQoL [41]. Our reference values provide a country-adapted basis because they were obtained from general population samples. Here, cut-off values of 56 for the UK and 65 for the NL should be taken to identify impaired TBI-specific HRQoL when comparing individuals after TBI with healthy individuals. As we found significant differences between the countries, we strongly recommend using the respective population-based reference values presented in the current study when the QOLIBRI is applied.

### Strengths and Limitations

A strength of the present study is the large size of the general population samples, which allowed for high-powered statistical analyses. The stratification into healthy individuals and those having reported at least one chronic health complaint offers an additional possibility for the interpretation of HRQoL of individuals after TBI.

The representativity of the recruited samples may be questioned. First, the selection of participants was based on different web-based panels. This might have led to different selection biases, even when several platforms were used for recruiting in order to increase the representativity of different groups. Second, no information was available from the survey agency concerning participants who were contacted but did not take part in the survey. In other words, it was not possible to determine how many and which individuals could have potentially participated in the study, as a means of demonstrating a selection bias. Third, answers given in the online survey could be associated with (self-)selection and nonresponse bias [42,43], as some individuals may systematically participate in online surveys.

Yet, the sampling procedure was strictly based on demographic characteristics such as age, sex, and education, and on a very large panel involving individuals from different sources. The quota sampling with respect to age, sex, and level of education corresponded to the distribution in the general populations of the two countries (see OECD statistics [35]). Therefore, the samples seem valid for providing reference values to evaluate the degree of impairment of HRQoL in individuals after TBI.

Another limitation was the lack of precise information concerning previously experienced TBIs in the general population samples. However, the estimated TBI prevalence based on reported age-adjusted hospital discharge rates due to TBI is quite low and reaches 312.7 per 100,000 in the UK and 173.7 per 100,000 in the NL [44]. Thus, the presence of individuals, who experienced TBI in the general population samples, was very unlikely to cause a bias concerning the reference values and evaluation of HRQoL.

The baseline characteristics of the general and the TBI sample displayed differences with regard to sex, age, and education status. However, such differences are unavoidable bearing in mind the two times higher prevalence of TBI among males [45] and increasing TBI incidence in elderly people [46], resulting in differences in work status distribution, and in higher rates of retired individuals in the TBI samples.

Furthermore, the relatively small sizes of the TBI samples required dichotomization of the QOLIBRI response categories for MI testing, which is associated with a loss of information concerning response patterns. However, the TBI sample was only used to ensure the methodological comparability of the QOLIBRI in general population samples by MI analyses. It turned out that the factorial structure and the understanding of the HRQoL construct measured by the QOLIBRI were comparable between the general population samples and the TBI samples in both countries. Thus, the reference values established here are reliable.

## 5. Conclusions

This paper aimed to provide a basis for a better understanding of HRQoL after TBI in research and clinical practice. For this purpose, population-based reference values were developed to add value to the interpretation and clinical meaningfulness of QOLIBRI scores of individuals after TBI. Significant differences in the reported levels of HRQoL were found between the UK and the NL general population samples as well as between the TBI and the general population samples. Therefore, we have presented population-based reference values separately for the two countries. We recommend establishing population-based reference values also for other countries in future research, especially for lower-income countries, as these are a key component for understanding therapeutic progress in individual cases and enabling research on HRQoL.

## Figures and Tables

**Table 1:** Sociodemographic and health-related data.

	UK			NL		
	Gen. Pop. Sample	TBI Sample		Gen. Pop. Sample	TBI Sample	
	*N* = 4403	*N* = 228	*p*	*N* = 3399	*N* = 432	*p*
**Age in years**						
mean ± SD	44.52 ± 15.66	49.73 ± 17.79	<0.001	45.2 ± 15.3	55.4 ± 18.8	<0.001
**Age category**						
18–40	1885 (42.8%)	67 (29.4%)	<0.001	1338 (39.4%)	98 (22.7%)	<0.001
41–64	1954 (44.4%)	113 (49.6%)		1651 (49.6%)	175 (40.5%)	
65+	564 (12.8%)	48 (21.1%)		410 (11.0%)	159 (36.8%)	
**Gender**						
Male	2134 (48.5%)	152 (66.7%)	<0.001	1665 (49.0%)	253 (58.6%)	<0.001
Female	2269 (51.5%)	76 (33.6%)		1734 (51.0%)	179 (41.4%)	
**Educational level**						
Low	1002 (22.8%)	7 (3.1%)	<0.001	1024 (30.1%)	14 (3.3%)	<0.001
Middle	1884 (42.8%)	99 (43.4%)		1526 (44.9%)	239 (55.3%)	
High	1517 (34.5%)	98 (43%)		849 (25.0%)	137 (31.7%)	
NA		24 (10.5%)			42 (9.7%)	
**Work status (before TBI)**						
In work	2267 (51.5%)	145 (63.6%)	<0.001	1776 (52.3%)	202 (46.8%)	<0.001
Out of work	399 (9.0%)	7 (3.1%)		374 (11.0%)	12 (2.8%)	
Looking after others	305 (6.9%)	1 (0.4%)		145 (4.3%)	7 (1.6%)	
Student	265 (6.0%)	10 (4.4%)		223 (6.6%)	34 (7.9%)	
Retired	725 (16.5%)	50 (21.9%)		446 (13.1%)	143 (33.1%)	
Unable to work	442 (10.0%)	3 (1.3%)		435 (12.8%)	7 (1.6%)	
NA		12 (5.3%)			27 (6.2%)	
Type of chronic health condition *						
Asthma	602 (13.0%)	-	-	336 (9.4%)	-	-
Heart disease	109 (2.3%)	-		102 (2.9%)	-	
Stroke	74 (1.6%)	-		81 (2.3%)	-	
Diabetes	390 (8.4%)	-		274 (7.7%)	-	
Back conditions	567 (12.2%)	-		355 (10.0%)	-	
Arthrosis	141 (3.0%)	-		346 (9.7%)	-	
Rheumatisms	192 (4.1%)	-		218 (6.1%)	-	
Cancer	128 (2.8%)	-		140 (3.9%)	-	
Memory problems (dementia)	82 (1.8%)	-		94 (2.6%)	-	
Memory problems (aging)	205 (4.4%)	-		82 (2.3%)	-	
Depression	1254 (27%)	-		423 (11.9%)	-	
Other	493 (10.6%)	-		628 (19.3%)	-	
**Number of chronic health conditions**						
None	2016 (45.8%)	-	-	1572 (46.2%)	-	-
One	1379 (31.3%)	-		1088 (32.0%)	-	
Two and more	1008 (22.9%)	-		739 (21.8%)	-	
**TBI-severity (GCS)**						
Mild	-	164 (71.9%)	-	-	366 (84.7%)	-
Moderate	-	7 (3.1%)		-	27 (6.3%)	
Severe	-	51 (22.4%)		-	29 (6.7%)	
NA	-	6 (2.6%)		-	10 (2.3%)	
**Recovery status (GOSE) at 3 months postinjury**						
Good recovery	-	111 (48.7%)	-	-	286 (66.2%)	-
Moderate disability	-	68 (29.8%)		-	107 (24.8%)	
Severe disability	-	47 (20.6%)		-	38 (8.8%)	
NA	-	2 (0.9%)		-	1 (0.2%)	

* Type of chronic health condition: multiple answers were allowed, therefore percentages were calculated separately for each complaint based on the total sample size; Note: UK: the United Kingdom; NL: the Netherlands; Gen. pop.: general population sample; TBI: TBI sample; *p*: *p*-value obtained with independent samples *t*-test for age or with chi-square test with Yates correction for gender, educational level, and work status; 65+: general population sample: 65–75, TBI-sample: 65–95; -: when data was not assessed; GCS: Glasgow Coma Scale; GOSE: Glasgow Outcome Scale Extended; In work: general population sample: employee and self-employed, TBI-sample: 35+ h/week and 20–34 h/week and <20 h/week and currently on sick leave and special employment; Out of work: general population sample: for more than 1 year and less than 1 year, TBI-Sample: unemployed; Housekeeper: general population sample: looking after others, e.g., kids or parents; Education level: TBI-sample: “low”: currently in school and primary school, “middle”: currently in diploma and secondary school/high school and post-high school, “high”: college/university.

**Table 2:** Results of the multiple regression analyses (total sample, UK, and the NL).

		Total Sample		UK		NL	
Predictors and Interactions	Reference Group	Β	*p*	β	*p*	β	*p*
NL	UK	8.76	**<0.001**	-	-	-	-
Age (41–64)	Age (18–40)	7.57	**<0.001**	9.36	**<0.001**	5.41	**<0.001**
Age (65–75)		13.11	**<0.001**	15.26	**<0.001**	9.91	**<0.001**
Sex (female)	Sex (male)	0.63	0.257	0.67	0.393	0.13	0.863
Education (middle)	Education (low)	3.07	**<0.001**	2.55	**<0.001**	3.57	**<0.001**
Education (high)		5.30	**<0.001**	5.35	**<0.001**	5.35	**<0.001**
Chronic health conditions (yes)	Chronic health conditions (no)	−16.38	**<0.001**	−16.70	**<0.001**	−15.88	**<0.001**
Age (41–64) × Chronic health conditions (yes)	Age (18–40) × Chronic health conditions (no)	−0.43	0.598	−2.80	**0.015**	2.02	0.066
Age (65–75) × Chronic health conditions (yes)		4.89	**<0.001**	4.91	**0.004**	4.98	**0.004**
Sex (female) × Chronic health conditions (yes)	Sex (male) × Chronic health conditions (no)	−0.19	0.805	−2.05	0.057	2.58	**0.012**

Note: β: regression coefficient; *p*: *p*-value; bold: *p*-values are significant on α = 0.05.

**Table 3:** Reference values for the Quality of Life after Traumatic Brain Injury (QOLIBRI) total score obtained from the general population UK sample stratified by sex, health status, age, and education.

**Sex × Health status × Age**				**Low HRQoL**		**−1 *SD***			***Md***			**+1 *SD***	**High HRQoL**	
**Sex**	**Health Status**	**Age**	***N***	**2.5%**	**5%**	**16%**	**30%**	**40%**	**50%**	**60%**	**70%**	**85%**	**95%**	**97.25%**
Female	Healthy	Age: 18–40	434	40	43	51	61	65	71	75	80	88	96	99
		Age: 41–64	408	49	50	60	71	76	80	84	88	94	100	100
		Age: 65–75	119	51	55	69	79	83	86	90	92	96	99	100
	At least one chronic condition	Age: 18–40	547	16	20	33	42	46	50	56	61	71	81	87
		Age: 41–64	587	12	19	36	46	50	55	61	68	79	90	94
		Age: 65–75	174	31	38	50	63	66	71	76	81	88	96	99
Male	Healthy	Age: 18–40	497	40	46	51	57	63	67	73	78	86	95	99
		Age: 41–64	442	49	50	59	70	75	80	83	87	95	100	100
		Age: 65–75	116	54	61	72	79	83	85	88	91	96	100	100
	At least one chronic condition	Age: 18–40	407	18	23	36	44	48	50	54	58	70	83	89
		Age: 41–64	517	14	19	36	46	52	57	64	71	83	93	98
		Age: 65–75	155	29	39	52	62	68	72	76	82	90	97	98
**Sex × Health status × Education**				**Low HRQoL**		**−1 *SD***			***Md***			**+1 *SD***	**High HRQoL**	
**Sex**	**Health Status**	**Education**	***N***	**2.5%**	**5%**	**16%**	**30%**	**40%**	**50%**	**60%**	**70%**	**85%**	**95%**	**97.25%**
Female	Healthy	education: low	193	44	49	54	65	71	77	81	85	93	97	100
		education: middle	383	46	49	57	67	73	79	82	87	94	100	100
		education: high	385	43	48	57	66	72	76	80	86	92	98	100
	At least one chronic condition	education: low	332	17	23	36	45	50	55	61	67	79	91	96
		education: middle	526	11	19	34	45	49	54	60	66	76	89	92
		education: high	450	16	21	38	45	50	56	62	69	78	87	93
Male	Healthy	education: low	197	41	46	54	61	71	78	81	85	95	100	100
		education: middle	493	45	49	54	63	69	74	79	83	91	99	100
		education: high	365	47	50	57	66	72	78	81	84	92	98	100
	At least one chronic condition	education: low	280	17	21	33	46	50	53	59	66	78	93	97
		education: middle	482	15	20	36	45	50	54	61	69	82	92	96
		education: high	317	25	28	44	50	54	58	63	71	83	92	97
		Total	4403	20	28	44	52	58	65	71	78	88	96	99

Note: HRQoL: health-related quality of life; 50% percentiles represent 50% of the distribution corresponding to the median (*Md*); *SD*: standard deviation; values from −1 standard deviation (16%) to +1 standard deviation (85%) are within the permissible range (i.e., not impaired HRQoL). Values below 16% (no symbols) indicate impaired HRQoL and values above 85% indicate outstanding HRQoL.

**Table 4:** Reference values for the QOLIBRI total score obtained from the general population NL sample stratified by sex, health status, age, and education.

**Sex × Health Status × Age**				**Low HRQoL**		**−1 *SD***			***Md***			**+1 *SD***	**High HRQoL**	
**Sex**	**Health status**	**Age**	**N**	**2.5%**	**5%**	**16%**	**30%**	**40%**	**50%**	**60%**	**70%**	**85%**	**95%**	**97.25%**
Female	Healthy	Age: 18–40	338	50	52	63	71	75	79	83	86	92	98	100
		Age: 41–64	292	50	58	69	75	79	83	86	90	96	100	100
		Age: 65–75	66	61	61	75	79	81	84	88	90	96	98	99
	At least one chronic condition	Age: 18–40	364	32	37	49	55	60	63	68	73	81	87	92
		Age: 41–64	527	38	44	54	62	66	71	75	79	87	94	96
		Age: 65–75	147	47	52	63	69	73	75	80	83	88	93	94
Male	Healthy	Age: 18–40	388	49	50	57	69	74	77	81	86	94	100	100
		Age: 41–64	396	53	56	68	75	79	83	89	92	96	100	100
		Age: 65–75	92	65	73	77	81	84	88	91	93	96	99	100
	At least one chronic condition	Age: 18–40	248	30	38	48	52	54	57	60	67	77	88	91
		Age: 41–64	436	31	37	50	58	63	69	73	77	86	95	98
		Age: 65–75	105	47	51	61	69	75	80	83	86	92	96	97
**Sex × Health Status × Education**				**Low HRQoL**		**−1 *SD***			***Md***			**+1 *SD***	**High HRQoL**	
**Sex**	**Health status**	**Education**	**N**	**2.5%**	**5%**	**16%**	**30%**	**40%**	**50%**	**60%**	**70%**	**85%**	**95%**	**97.25%**
Female	Healthy	education: low	171	49	50	61	70	75	79	82	86	94	99	100
		education: middle	341	50	56	68	75	78	81	84	88	95	100	100
		education: high	184	51	59	69	75	79	84	86	88	93	98	99
	At least one chronic condition	education: low	374	34	41	52	59	65	68	73	78	84	91	96
		education: middle	477	34	42	52	60	64	69	73	78	85	92	96
		education: high	187	43	48	54	63	67	71	76	80	86	92	95
Male	Healthy	education: low	202	50	50	60	71	76	79	82	88	95	99	100
		education: middle	394	50	54	65	74	77	81	85	92	96	100	100
		education: high	280	50	52	66	75	79	83	88	91	96	100	100
	At least one chronic condition	education: low	277	30	35	50	57	61	66	70	75	83	93	96
		education: middle	314	32	41	49	56	59	66	72	77	86	96	98
		education: high	198	36	42	50	57	62	68	72	79	87	92	95
		Total	3399	39	46	55	65	71	75	79	83	92	98	100

Note: HRQoL: health-related quality of life; 50% percentiles represent 50% of the distribution corresponding to the median (*Md*); *SD*: standard deviation; values from −1 standard deviation (16%) to +1 standard deviation (85%) are within the normal range (i.e., not impaired HRQoL); Values below 16% indicate impaired HRQoL and values above 85% indicate outstanding HRQoL.

## Supplementary Materials

The following are available online at https://www.mdpi.com/2077-0383/9/7/2100/s1, Table S1: Reference values for the QOLIBRI Cognition scale (UK), Table S2: Reference values for the QOLIBRI Cognition scale (NL).

## Appendix A

**Table A1:** Characteristics of the QOLIBRI items.

General Population Sample								
	UK	NL	UK	NL	UK	NL	UK	NL
QOLIBRI Items	Mean ± SD		Skewness		Ceiling (%)		% “Not at All” and “Slightly”	
Cognition	71.1 ± 24.4	78.4 ± 18.1	−0.8	−0.9	15.1	16.8	-	-
Concentrate	3.7 ± 1.2	4.0 ± 0.9	−0.6	−1	30.4	34.5	17	7
Express yourself	3.8 ± 1.1	4.1 ± 0.9	−0.8	−1.1	35.2	38	13	6
Memory	3.7 ± 1.1	4.1 ± 0.9	−0.6	−1	26.8	33.8	15	6
Plan and problem solve	3.9 ± 1.1	4.2 ± 0.9	−0.8	−1.2	35	41	12	5
Decisions	3.9 ± 1.1	4.1 ± 0.9	−0.9	−1.1	38.6	39	11	5
Navigate	4.1 ± 1.1	4.2 ± 0.9	−1	−1.2	46.2	44.7	10	4
Speed of thinking	3.9 ± 1.1	4.2 ± 0.9	−0.8	−1.2	32.7	39.6	12	5
Self	54.8 ± 27.4	68.6 ± 20.3	−0.2	−0.7	6.2	6.4	-	-
Energy	3.2 ± 1.2	3.5 ± 1.0	−0.3	−0.6	13	15.1	27	15
Motivation	3.2 ± 1.2	3.7 ± 1.0	−0.3	−0.7	15.4	21.2	26	11
Self-esteem	3.2 ± 1.3	3.8 ± 1.0	−0.2	−0.8	17.2	28.2	30	11
Appearance	3.1 ± 1.2	3.8 ± 1.0	−0.2	−0.8	13.4	20.2	31	10
Achievements	3.2 ± 1.2	3.8 ± 1.0	−0.2	−0.8	16.2	25.8	29	11
Self-perception	3.2 ± 1.2	3.8 ± 1.0	−0.3	−0.9	14.8	21.7	28	11
Future	3.2 ± 1.2	3.7 ± 1.0	−0.2	−0.8	15.4	21.5	29	12
Daily life and autonomy	66.5 ± 26.2	75.5 ± 19.4	−0.6	−0.8	11.9	13.2	-	-
Independence	3.7 ± 1.2	4.1 ± 1.0	−0.7	−1	33.3	38.4	16	7
Get out and about	3.8 ± 1.2	4.2 ± 0.9	−0.8	−1.3	40.2	45.3	16	5
Domestic activities	3.9 ± 1.2	4.1 ± 1.0	−0.8	−1.1	40.7	42.5	15	7
Run personal finances	3.8 ± 1.2	4.2 ± 0.9	−0.8	−1.2	36.7	43.5	16	6
Participation work	3.5 ± 1.3	3.8 ± 1.2	−0.5	−0.9	27.4	30.6	22	15
Social and leisure activities	3.3 ± 1.3	3.8 ± 1.1	−0.3	−0.8	21.5	26.9	28	13
In charge of life	3.6 ± 1.2	4.0 ± 0.9	−0.6	−1	28.8	35	18	7
Social relationships	63.9 ± 26.0	74.0 ± 19.6	−0.5	−0.8	11	11.5	-	-
Affection towards others	3.8 ± 1.2	4.2 ± 0.9	−0.7	−1.2	34	43.9	15	6
Family	3.8 ± 1.2	4.0 ± 1.0	−0.7	−1	33.8	35.9	15	8
Friends	3.6 ± 1.2	4.0 ± 0.9	−0.6	−1.1	28.8	34.9	18	7
Partner	3.7 ± 1.3	4.0 ± 1.1	−0.7	−1.1	37.1	43	19	10
Sex life	3.0 ± 1.4	3.5 ± 1.2	−0.1	−0.7	20.6	23.9	35	19
Attitudes of others	3.4 ± 1.1	3.9 ± 0.9	−0.4	−0.9	19.7	26.4	19	7
Emotions	59.4 ± 28.0	68.4 ± 24.4	−0.2	−0.4	11.6	15.4	-	-
Feel lonely	3.5 ± 1.3	3.5 ± 1.3	−0.4	−0.3	32.1	29.2	24	25
Feel bored	3.4 ± 1.3	3.6 ± 1.2	−0.3	−0.5	26.2	28.2	26	20
Feel anxious	3.3 ± 1.4	3.7 ± 1.2	−0.2	−0.6	25.1	35.2	32	15
Feel sad	3.3 ± 1.4	3.7 ± 1.3	−0.2	−0.6	27	35.5	31	18
Feel angry	3.6 ± 1.3	3.9 ± 1.2	−0.5	−0.8	34.7	41.6	22	13
Physical problems	66.8 ± 27.0	70.0 ± 23.5	−0.5	−0.5	15.6	15.3	-	-
Slow/clumsiness	3.8 ± 1.2	3.9 ± 1.1	−0.7	−0.7	40.8	38.8	17	14
Effects injuries	3.9 ± 1.2	3.9 ± 1.2	−0.8	−0.9	46.4	47.2	15	16
Pain	3.5 ± 1.3	3.6 ± 1.2	−0.4	−0.4	28.6	28.8	23	22
See/hear	4.0 ± 1.2	4.0 ± 1.1	−0.9	−0.8	46	40.1	14	11
Effects health problems	3.5 ± 1.3	3.7 ± 1.2	−0.4	−0.5	28.8	31.1	23	19
QOLIBRI total score	63.8 ± 20.6	72.8 ± 16.6	−0.3	−0.5	1.6	1.9	-	-

Note: Mean: mean value; SD: standard deviation; ceiling effects are expressed as a percentage and represent the proportion of individuals who chose the response category “very” on the QOLIBRI items or reached the maximum of 100 on the respective QOLIBRI scales.

**Table A2:** Psychometric properties of the QOLIBRI scales.

	Cronbach’s Alpha	Item-Total Correlation Range	Correlations between Subscales Scores				
**UK general population sample**							
**QOLIBRI domain**			**(1)**	**(2)**	**(3)**	**(4)**	**(5)**
Cognition (1)	0.94	0.77–0.83	1				
Self (2)	0.95	0.76–0.87	0.67	1			
Daily life and autonomy (3)	0.93	0.71–0.81	0.75	0.77	1		
Social relationships (4)	0.9	0.60–0.76	0.65	0.73	0.71	1	
Emotions (5)	0.87	0.62–0.79	0.46	0.51	0.49	0.47	1
Physical problems (6)	0.88	0.61–0.74	0.5	0.44	0.55	0.39	0.48
**NL general population sample**							
**QOLIBRI domain**			**(1)**	**(2)**	**(3)**	**(4)**	**(5)**
Cognition (1)	0.92	0.69–0.77	1				
Self (2)	0.92	0.70–0.81	0.68	1			
Daily life and autonomy (3)	0.89	0.61–0.73	0.7	0.76	1		
Social relationships (4)	0.86	0.60–0.72	0.58	0.66	0.66	1	
Emotions (5)	0.88	0.72–0.82	0.54	0.57	0.56	0.55	1
Physical problems (6)	0.86	0.54–0.78	0.52	0.57	0.62	0.45	0.49

**Table A3:** Results of confirmatory factor analyses.

**UK General Population Sample**							
					**Model Comparison**		
**Model with Factors**	**CFI**	**RMSEA (90%CI)**	**χ^2^ (df)**	***p***	**Model with Factors**	**Δχ^2^ (Δdf)**	***p***
one	0.802	0.156 (0.155; 0.157)	68,302 (629)	<0.001			
two	0.889	0.117 (0.116; 0.118)	38,601 (628)	<0.001	one vs. two	3111.5 (1)	<0.001
six	0.957	0.074 (0.073; 0.075)	15,441 (614)	<0.001	two vs. six	5696.1 (14)	<0.001
**NL General Population Sample**							
					**Model Comparison**		
**Model with Factors**	**CFI**	**RMSEA (90%CI)**	**χ^2^(df)**	***p***	**Model with Factors**	**Δχ^2^(Δdf)**	***p***
one	0.8	0.137 (0.136; 0.138)	40,659 (629)	<0.001			
two	0.868	0.111 (0.110; 0.113)	27,135 (628)	<0.001	one vs. two	1725.3 (1)	<0.001
six	0.952	0.068 (0.067; 0.069)	10,276 (614)	<0.001	two vs. six	4313.8 (14)	<0.001

Note: CFI: scaled Comparative Fit Index; RMSEA (90%CI): scaled root mean square error of approximation with 90% confidence interval; χ^2^: scaled chi-square statistics; df: scaled degrees of freedom; *p*: *p*-value of chi-square (difference) statistics; Δχ^2^: difference in chi-square statistics under Sattora.Bentler.2001 correction; Δdf: difference in degrees of freedom.

**Table A4:** Results of measurement invariance testing.

**UK: General Population Sample vs. TBI Sample**						
				**Model Comparison**		
**CFI**	**RMSEA (90%CI)**	**χ^2^ (df)**	***p***	**Invariance Models**	**Δχ^2^ (Δdf)**	***P***
0.989	0.033 (0.032; 0.034)	4264 (1228)	<0.001			
0.991	0.029 (0.028; 0.031)	4740 (1253)	<0.001	Configural vs. partial	23.00 (25)	0.577
0.991	0.029 (0.028; 0.030)	4854 (1290)	<0.001	Partial vs. full	66.95 (37)	0.002
**NL: General Population Sample vs. TBI Sample**						
				**Model Comparison**		
**CFI**	**RMSEA (90%CI)**	**χ^2^ (df)**	***p***	**Invariance Models**	**Δχ^2^ (Δdf)**	***P***
0.983	0.032 (0.031; 0.034)	3409 (1228)	<0.001			
0.986	0.029 (0.027; 0.030)	3544 (1253)	<0.001	Configural vs. partial	8.27 (25)	0.999
0.986	0.029 (0.028; 0.030)	3702 (1290)	<0.001	Partial vs. full	108.10 (37)	<0.001
**UK vs. NL: General Population Samples**						
				**Model Comparison**		
**CFI**	**RMSEA (90%CI)**	**χ^2^ (df)**	***p***	**Invariance Models**	**Δχ^2^ (Δdf)**	***P***
0.956	0.071 (0.071; 0.072)	16696 (1228)	<0.001			
0.966	0.062 (0.061; 0.063)	17884 (2153)	<0.001	Configural vs. partial	87.27 (25)	<0.001
0.962	0.062 (0.061; 0.063)	20051 (1410)	<0.001	Partial vs. full	2395.26 (148)	<0.001

Note: CFI: scaled Comparative Fit Index; RMSEA (90%CI): scaled root mean square error of approximation with 90% confidence interval; χ^2^: scaled chi-square statistics; df: scaled degrees of freedom; *p*: *p*-value of chi-square (difference) statistics; Δχ^2^: difference in chi-square statistics under Sattora-Bentler (2001) correction; Δdf: difference in degrees of freedom; Identification constraints for the invariance models: Configural: item intercepts = 0, residual variances = 1, latent factor means = 0, latent factor variances = 1 Partial: item intercepts = 0, residual variances = 1. Only in the reference group latent factor means = 0 and variances = 1; Full: item intercepts = 0, residual variances = 1. Only in the reference group factor means = 0, factor variances = 1.

**Table A5:** Response probabilities (RP) of the UK and NL general population samples to choose a response category estimated in the partial invariance model and their differences to the response probabilities estimated within the full invariance model.

	UK Gen. Pop. (TBI as a Ref.) ^a^	NL Gen. Pop. (TBI as a Ref.) ^b^	NL Gen. Pop. (UK as a Reference) ^c^				
**Cognition**	**1 ^d^**	**1 ^d^**	**Not at All**	**Slightly**	**Moderately**	**Quite**	**Very**
Concentrate	0.614 (0.001)	0.777 (0.001)	0.050 (−0.001)	0.124 (0.012)	0.213 (0.014)	0.309 (−0.039)	0.304 (0.013)
Express yourself	0.670 (−0.003)	0.814 (−0.004)	0.038 (−0.001)	0.097 (0.005)	0.195 (0.013)	0.318 (−0.041)	0.352 (0.023)
Memory	0.624 (0.004)	0.793 (0.004)	0.042 (−0.001)	0.109 (0.009)	0.225 (0.022)	0.355 (−0.027)	0.268 (−0.003)
Plan and problem solve	0.685 (0.001)	0.841 (−0.001)	0.033 (−0.001)	0.086 (0.005)	0.196 (0.020)	0.334 (−0.035)	0.350 (0.011)
Decisions	0.705 (0.000)	0.824 (−0.001)	0.034 (−0.004)	0.080 (0.001)	0.181 (0.008)	0.319 (−0.043)	0.386 (0.038)
Navigate	0.747 (−0.004)	0.843 (−0.005)	0.026 (−0.002)	0.071 (0.001)	0.157 (0.001)	0.285 (−0.049)	0.462 (0.049)
Speed of thinking	0.677 (0.003)	0.836 (0.006)	0.032 (−0.004)	0.086 (0.007)	0.205 (0.022)	0.350 (−0.029)	0.327 (0.005)
**Self**							
Energy	0.431 (0.006)	0.589 (0.007)	0.116 (−0.014)	0.157 (−0.012)	0.296 (0.010)	0.300 (−0.005)	0.130 (0.020)
Motivation	0.448 (0.003)	0.667 (0.008)	0.109 (−0.003)	0.156 (−0.006)	0.288 (0.017)	0.294 (−0.021)	0.154 (0.013)
Self-esteem	0.438 (0.001)	0.696 (0.000)	0.129 (−0.003)	0.173 (0.006)	0.260 (0.020)	0.266 (−0.022)	0.172 (0.000)
Appearance	0.409 (−0.001)	0.69 (−0.008)	0.136 (0.004)	0.178 (0.018)	0.277 (0.022)	0.275 (−0.048)	0.134 (0.004)
Achievements	0.438 (−0.006)	0.69 (−0.003)	0.120 (−0.006)	0.166 (0.005)	0.275 (0.025)	0.276 (−0.025)	0.162 (0.001)
Self-perception	0.443 (−0.001)	0.701 (−0.003)	0.118 (−0.007)	0.166 (0.006)	0.273 (0.024)	0.295 (−0.031)	0.148 (0.008)
Future	0.433 (−0.002)	0.677 (−0.001)	0.123 (−0.008)	0.163 (0.005)	0.282 (0.025)	0.279 (−0.033)	0.154 (0.011)
**Daily life & Autonomy**							
Independence	0.623 (−0.003)	0.771 (−0.003)	0.056 (−0.003)	0.105 (0.005)	0.216 (0.009)	0.290 (−0.028)	0.333 (0.017)
Get out and about	0.655 (0.003)	0.836 (−0.001)	0.060 (0.001)	0.100 (0.012)	0.185 (0.014)	0.253 (−0.052)	0.402 (0.025)
Domestic activities	0.672 (−0.001)	0.791 (−0.001)	0.052 (−0.004)	0.095 (0.002)	0.182 (−0.001)	0.265 (−0.036)	0.407 (0.039)
Run personal finances	0.650 (−0.003)	0.81 (−0.004)	0.057 (0.000)	0.099 (0.009)	0.194 (0.013)	0.283 (−0.033)	0.367 (0.011)
Participation work	0.554 (0.003)	0.677 (0.007)	0.115 (−0.012)	0.100 (−0.010)	0.231 (0.022)	0.280 (−0.018)	0.274 (0.017)
Social and leisure activities	0.472 (0.001)	0.662 (0.005)	0.129 (0.007)	0.153 (0.010)	0.246 (0.016)	0.257 (−0.034)	0.215 (0.002)
In charge of life	0.584 (0.000)	0.773 (−0.001)	0.069 (−0.003)	0.114 (0.009)	0.233 (0.018)	0.296 (−0.033)	0.288 (0.010)
**Social relationships**							
Affection towards others	0.627 (−0.002)	0.806 (−0.001)	0.050 (−0.004)	0.105 (0.008)	0.218 (0.012)	0.287 (−0.030)	0.340 (0.014)
Family	0.633 (−0.001)	0.758 (−0.005)	0.057 (−0.007)	0.096 (−0.003)	0.213 (0.003)	0.295 (−0.035)	0.338 (0.042)
Friends	0.593 (−0.002)	0.784 (−0.002)	0.072 (0.000)	0.110 (0.007)	0.225 (0.013)	0.305 (−0.044)	0.288 (0.023)
Partner	0.615 (0.000)	0.758 (0.000)	0.102 (−0.001)	0.092 (−0.001)	0.191 (0.005)	0.244 (−0.032)	0.371 (0.028)
Sex life	0.425 (0.005)	0.591 (0.010)	0.234 (0.021)	0.120 (0.001)	0.221 (0.000)	0.218 (−0.039)	0.206 (0.017)
Attitudes of others	0.517 (0.000)	0.766 (0.000)	0.070 (0.001)	0.124 (0.010)	0.289 (0.031)	0.320 (−0.053)	0.197 (0.010)
**Emotions**							
Feel lonely	0.529 (−0.004)	0.534 (−0.010)	0.079 (−0.003)	0.160 (−0.035)	0.232 (−0.002)	0.208 (−0.006)	0.321(0.046)
Feel bored	0.494 (0.000)	0.589 (−0.003)	0.089 (0.006)	0.171 (−0.010)	0.245 (0.002)	0.232 (−0.023)	0.262(0.026)
Feel anxious	0.463 (0.002)	0.633 (−0.001)	0.136 (0.022)	0.181 (0.008)	0.219 (−0.010)	0.212 (−0.021)	0.251 (0.001)
Feel sad	0.474 (0.003)	0.620 (0.010)	0.138 (0.018)	0.171 (−0.007)	0.217 (−0.005)	0.204 (−0.018)	0.270 (0.011)
Feel angry	0.567 (−0.002)	0.695 (0.002)	0.082 (0.008)	0.136 (−0.001)	0.214 (0.003)	0.22 (−0.022)	0.347 (0.012)
**Physical problems**							
Slow/clumsiness	0.612 (0.002)	0.649 (0.002)	0.055 (0.008)	0.118 (−0.005)	0.214 (0.003)	0.204 (−0.021)	0.408 (0.015)
Effects injuries	0.658 (0.005)	0.679 (0.013)	0.048 (−0.004)	0.105 (−0.011)	0.189 (0.014)	0.194 (−0.003)	0.464 (0.004)
Pain	0.545 (−0.002)	0.542 (−0.010)	0.088 (0.006)	0.146 (−0.010)	0.221 (−0.007)	0.260 (0.009)	0.286 (0.002)
See/hear	0.674 (0.000)	0.688 (0.002)	0.041 (0.004)	0.098 (0.001)	0.187 (−0.008)	0.215 (−0.029)	0.460 (0.032)
Effects health problems	0.534 (−0.004)	0.595 (−0.006)	0.090 (0.011)	0.143 (−0.004)	0.233 (0.009)	0.245 (−0.010)	0.288 (−0.007)

Note. ^a^ Probabilities estimated for the NL general population sample from the invariance model comparing TBI and general population sample; ^b^ Probabilities estimated for the UK general population sample from the invariance model comparing TBI and general population sample; ^c^ Probabilities estimated for the NL general population sample from the invariance model comparing UK and NL general population samples; ^d^ For measurement invariance testing with TBI samples response categories “not at all” and “slightly” were recorded as 1.

## Appendix B. The CENTER-TBI Participants and Investigators

Cecilia Åkerlund ^1^, Krisztina Amrein ^2^, Nada Andelic ^3^, Lasse Andreassen ^4^, Audny Anke ^5^, Anna Antoni ^6^, Gérard Audibert ^7^, Philippe Azouvi ^8^, Maria Luisa Azzolini ^9^, Ronald Bartels ^10^, Pál Barzó ^11^, Romuald Beauvais ^12^, Ronny Beer ^13^, Bo-Michael Bellander ^14^, Antonio Belli ^15^, Habib Benali ^16^, Maurizio Berardino ^17^, Luigi Beretta ^9^, Morten Blaabjerg ^18^, Peter Bragge ^19^, Alexandra Brazinova ^20^, Vibeke Brinck ^21^, Joanne Brooker ^22^, Camilla Brorsson ^23^, Andras Buki ^24^, Monika Bullinger ^25^, Manuel Cabeleira ^26^, Alessio Caccioppola ^27^, Emiliana Calappi ^27^, Maria Rosa Calvi ^9^, Peter Cameron ^28^, Guillermo Carbayo Lozano ^29^, Marco Carbonara ^27^, Simona Cavallo ^17^, Giorgio Chevallard ^30^, Arturo Chieregato ^30^, Giuseppe Citerio ^31,32^, Iris Ceyisakar ^33^, Hans Clusmann ^34^, Mark Coburn ^35^, Jonathan Coles ^36^, Jamie D. Cooper ^37^, Marta Correia ^38^, Amra Čović ^39^, Nicola Curry ^40^, Endre Czeiter ^24^, Marek Czosnyka ^26^, Claire Dahyot-Fizelier ^41^, Paul Dark ^42^, Helen Dawes ^43^, Véronique De Keyser ^44^, Vincent Degos ^16^, Francesco Della Corte ^45^, Hugo den Boogert ^10^, Bart Depreitere ^46^, Đula Đilvesi ^47^, Abhishek Dixit ^48^, Emma Donoghue ^22^, Jens Dreier ^49^, Guy-Loup Dulière ^50^, Ari Ercole ^48^, Patrick Esser ^43^, Erzsébet Ezer ^51^, Martin Fabricius ^52^, Valery L. Feigin ^53^, Kelly Foks ^54^, Shirin Frisvold ^55^, Alex Furmanov ^56^, Pablo Gagliardo ^57^, Damien Galanaud ^16^, Dashiell Gantner ^28^, Guoyi Gao ^58^, Pradeep George ^59^, Alexandre Ghuysen ^60^, Lelde Giga ^61^, Ben Glocker ^62^, Jagoš Golubovic ^47^, Pedro A. Gomez ^63^, Johannes Gratz ^64^, Benjamin Gravesteijn ^33^, Francesca Grossi ^45^, Russell L. Gruen ^65^, Deepak Gupta ^66^, Juanita A. Haagsma ^33^, Iain Haitsma ^67^, Raimund Helbok ^13^, Eirik Helseth ^68^, Lindsay Horton ^69^, Jilske Huijben ^33^, Peter J. Hutchinson ^70^, Bram Jacobs ^71^, Stefan Jankowski ^72^, Mike Jarrett ^21^, Ji-yao Jiang ^58^, Faye Johnson ^73^, Kelly Jones ^53^, Mladen Karan ^47^, Angelos G. Kolias ^70^, Erwin Kompanje ^74^, Daniel Kondziella ^52^, Evgenios Koraropoulos ^48^, Lars-Owe Koskinen ^75^, Noémi Kovács ^76^, Ana Kowark ^35^, Alfonso Lagares ^63^, Linda Lanyon ^59^, Steven Laureys ^77^, Fiona Lecky ^78,79^, Didier Ledoux ^77^, Rolf Lefering ^80^, Valerie Legrand ^81^, Aurelie Lejeune ^82^, Leon Levi ^83^, Roger Lightfoot ^84^, Hester Lingsma ^33^, Andrew I.R. Maas ^44^, Ana M. Castaño-León ^63^, Marc Maegele ^85^, Marek Majdan ^20^, Alex Manara ^86^, Geoffrey Manley ^87^, Costanza Martino ^88^, Hugues Maréchal ^50^, Julia Mattern ^89^, Catherine McMahon ^90^, Béla Melegh ^91^, David Menon ^48^, Tomas Menovsky ^44^, Ana Mikolic ^33^, Benoit Misset ^77^, Visakh Muraleedharan ^59^, Lynnette Murray ^28^, Ancuta Negru ^92^, David Nelson ^1^, Virginia Newcombe ^48^, Daan Nieboer ^33^, József Nyirádi ^2^, Otesile Olubukola ^78^, Matej Oresic ^93^, Fabrizio Ortolano ^27^, Aarno Palotie ^94,95,96^, Paul M. Parizel ^97^, Jean-François Payen ^98^, Natascha Perera ^12^, Vincent Perlbarg ^16^, Paolo Persona ^99^, Wilco Peul ^100^, Anna Piippo-Karjalainen ^101^, Matti Pirinen ^94^, Horia Ples ^92^, Suzanne Polinder ^33^, Inigo Pomposo ^29^, Jussi P. Posti ^102^, Louis Puybasset ^103^, Andreea Radoi ^104^, Arminas Ragauskas ^105^, Rahul Raj ^101^, Malinka Rambadagalla ^106^, Jonathan Rhodes ^107^, Sylvia Richardson ^108^, Sophie Richter ^48^, Samuli Ripatti ^94^, Saulius Rocka ^105^, Cecilie Roe ^109^, Olav Roise ^110,111^, Jonathan Rosand ^112^, Jeffrey V. Rosenfeld ^113^, Christina Rosenlund ^114^, Guy Rosenthal ^56^, Rolf Rossaint ^35^, Sandra Rossi ^99^, Daniel Rueckert ^62^, Martin Rusnák ^115^, Juan Sahuquillo ^104^, Oliver Sakowitz ^89,116^, Renan Sanchez-Porras ^116^, Janos Sandor ^117^, Nadine Schäfer ^80^, Silke Schmidt ^118^, Herbert Schoechl ^119^, Guus Schoonman ^120^, Rico Frederik Schou ^121^, Elisabeth Schwendenwein ^6^, Charlie Sewalt ^33^, Toril Skandsen ^122,123^, Peter Smielewski ^26^, Abayomi Sorinola ^124^, Emmanuel Stamatakis ^48^, Simon Stanworth ^40^, Robert Stevens ^125^, William Stewart ^126^, Ewout W. Steyerberg ^33,127^, Nino Stocchetti ^128^, Nina Sundström ^129^, Anneliese Synnot ^22,130^, Riikka Takala ^131^, Viktória Tamás ^124^, Tomas Tamosuitis ^132^, Mark Steven Taylor ^20^, Braden Te Ao ^53^, Olli Tenovuo ^102^, Alice Theadom ^53^, Matt Thomas ^86^, Dick Tibboel ^133^, Marjolein Timmers ^74^, Christos Tolias ^134^, Tony Trapani ^28^, Cristina Maria Tudora ^92^, Peter Vajkoczy ^135^, Shirley Vallance ^28^, Egils Valeinis ^61^, Zoltán Vámos ^51^, Mathieu van der Jagt ^136^, Gregory Van der Steen ^44^, Joukje van der Naalt ^71^, Jeroen T.J.M. van Dijck ^100^, Thomas A. van Essen ^100^, Wim Van Hecke ^137^, Caroline van Heugten ^138^, Dominique Van Praag ^139^, Thijs Vande Vyvere ^137^, Roel P. J. van Wijk ^100^, Alessia Vargiolu ^32^, Emmanuel Vega ^82^, Kimberley Velt ^33^, Jan Verheyden ^137^, Paul M. Vespa ^140^, Anne Vik ^122,141^, Rimantas Vilcinis ^132^, Victor Volovici ^67^, Nicole von Steinbüchel ^39^, Daphne Voormolen ^33^, Petar Vulekovic ^47^, Kevin K.W. Wang ^142^, Eveline Wiegers ^33^, Guy Williams ^48^, Lindsay Wilson ^69^, Stefan Winzeck ^48^, Stefan Wolf ^143^, Zhihui Yang ^142^, Peter Ylén ^144^, Alexander Younsi ^89^, Frederick A. Zeiler ^48,145^, Veronika Zelinkova ^20^, Agate Ziverte ^61^, Tommaso Zoerle ^27^

^1^ Department of Physiology and Pharmacology, Section of Perioperative Medicine and Intensive Care, Karolinska Institutet, 17176, Stockholm, Sweden

^2^ János Szentágothai Research Centre, University of Pécs, 7622, Pécs, Hungary

^3^ Division of Surgery and Clinical Neuroscience, Department of Physical Medicine and Rehabilitation, Oslo University Hospital and University of Oslo, 0424, Oslo, Norway

^4^ Department of Neurosurgery, University Hospital Northern Norway, 9019, Tromso, Norway

^5^ Department of Physical Medicine and Rehabilitation, University Hospital Northern Norway, 9019, Tromso, Norway

^6^ Trauma Surgery, Medical University Vienna, 1090, Vienna, Austria

^7^ Department of Anesthesiology & Intensive Care, University Hospital Nancy, 54000, Nancy, France

^8^ Raymond Poincare hospital, Assistance Publique—Hopitaux de Paris, 75013, Paris, France

^9^ Department of Anesthesiology & Intensive Care, San Raffaele University Hospital, 20132, Milan, Italy

^10^ Department of Neurosurgery, Radboud University Medical Center, 6500 HB, Nijmegen, The Netherlands

^11^ Department of Neurosurgery, University of Szeged, 6720, Szeged, Hungary

^12^ International Projects Management, ARTTIC, 80333, Munchen, Germany

^13^ Department of Neurology, Neurological Intensive Care Unit, Medical University of Innsbruck, 6020, Innsbruck, Austria

^14^ Department of Neurosurgery & Anesthesia & intensive care medicine, Karolinska University Hospital, 17176, Stockholm, Sweden

^15^ NIHR Surgical Reconstruction and Microbiology Research Centre, B15 2TH, Birmingham, UK

^16^ Anesthesie-Réanimation, Assistance Publique—Hopitaux de Paris, 75013, Paris, France

^17^ Department of Anesthesia & ICU, AOU Città della Salute e della Scienza di Torino—Orthopedic and Trauma Center, 10126, Torino, Italy

^18^ Department of Neurology, Odense University Hospital, 5000, Odense, Denmark

^19^ BehaviourWorks Australia, Monash Sustainability Institute, Monash University, Victoria, Australia, 3004, Melbourne, Australia

^20^ Department of Public Health, Faculty of Health Sciences and Social Work, Trnava University, 91843, Trnava, Slovakia

^21^ Quesgen Systems Inc., Burlingame, CA 94010, California, CA, USA

^22^ Australian & New Zealand Intensive Care Research Centre, Department of Epidemiology and Preventive Medicine, School of Public Health and Preventive Medicine, Monash University, 3004, Melbourne, Australia

^23^ Department of Surgery and Perioperative Science, Umeå University, 90185, Umeå, Sweden

^24^ Department of Neurosurgery, Medical School, University of Pécs, Hungary and Neurotrauma Research Group, János Szentágothai Research Centre, University of Pécs, Hungary, 7622, Pécs, Hungary

^25^ Department of Medical Psychology, Universitätsklinikum Hamburg-Eppendorf, 20251, Hamburg, Germany

^26^ Brain Physics Lab, Division of Neurosurgery, Department of Clinical Neurosciences, University of Cambridge, Addenbrooke’s Hospital, CB2 0QQ, Cambridge, UK

^27^ Neuro ICU, Fondazione IRCCS Cà Granda Ospedale Maggiore Policlinico, 20122, Milan, Italy

^28^ ANZIC Research Centre, Department of Epidemiology and Preventive Medicine, Monash University, Melbourne, Victoria, Australia, 3004, Melbourne, Australia

^29^ Department of Neurosurgery, Hospital of Cruces, 48903, Bilbao, Spain

^30^ NeuroIntensive Care, Niguarda Hospital, 20122, Milan, Italy

^31^ School of Medicine and Surgery, Università Milano Bicocca, 20126, Milano, Italy

^32^ NeuroIntensive Care, ASST di Monza, 20900, Monza, Italy

^33^ Department of Public Health, Erasmus Medical Center-University Medical Center, Rotterdam, The Netherlands

^34^ Department of Neurosurgery, Medical Faculty RWTH Aachen University, 52074, Aachen, Germany

^35^ Department of Anaesthesiology, University Hospital of Aachen, 52074, Aachen, Germany

^36^ Department of Anesthesia & Neurointensive Care, Cambridge University Hospital NHS Foundation Trust, CB2 0QQ. Cambridge, UK

^37^ School of Public Health & PM, Monash University and The Alfred Hospital, Melbourne, Victoria, Australia, 3004, Melbourne, Australia

^38^ Radiology/MRI department, MRC Cognition and Brain Sciences Unit, CB 0QQ, Cambridge, UK

^39^ Institute of Medical Psychology and Medical Sociology, Universitätsmedizin Göttingen, 37073, Göttingen, Germany

^40^ Oxford University Hospitals NHS Trust, OX 0BP, Oxford, UK

^41^ Intensive Care Unit, CHU Poitiers, 86021, Potiers, France

^42^ Critical Care Directorate, Salford Royal Hospital NHS Foundation Trust, University of Manchester NIHR Biomedical Research Centre, M5 5AP, Salford, UK

^43^ Movement Science Group, Faculty of Health and Life Sciences, Oxford Brookes University, OX3 DU, Oxford, UK

^44^ Department of Neurosurgery, Antwerp University Hospital and University of Antwerp, 2650, Edegem, Belgium

^45^ Department of Anesthesia & Intensive Care, Maggiore Della Carità Hospital, 28100, Novara, Italy

^46^ Department of Neurosurgery, University Hospitals Leuven, 3000, Leuven, Belgium

^47^ Department of Neurosurgery, Clinical centre of Vojvodina, Faculty of Medicine, University of Novi Sad, 21000, Novi Sad, Serbia

^48^ Division of Anaesthesia, University of Cambridge, Addenbrooke’s Hospital, CB2 0QQ, Cambridge, UK

^49^ Center for Stroke Research Berlin, Charité—Universitätsmedizin Berlin, corporate member of Freie Universität Berlin, Humboldt-Universität zu Berlin, and Berlin Institute of Health, 13353, Berlin, Germany

^50^ Intensive Care Unit, CHR Citadelle, 4000, Liège, Belgium

^51^ Department of Anaesthesiology and Intensive Therapy, University of Pécs, 7622, Pécs, Hungary

^52^ Departments of Neurology, Clinical Neurophysiology and Neuroanesthesiology, Region Hovedstaden Rigshospitalet, 2100, Copenhagen, Denmark

^53^ National Institute for Stroke and Applied Neurosciences, Faculty of Health and Environmental Studies, Auckland University of Technology, 1010, Auckland, New Zealand

^54^ Department of Neurology, Erasmus MC, 3015 GD Rotterdam, the Netherlands

^55^ Department of Anesthesiology and Intensive care, University Hospital Northern Norway, 9019, Tromso, Norway

^56^ Department of Neurosurgery, Hadassah-hebrew University Medical center, 91120, Jerusalem, Israel

^57^ Fundación Instituto Valenciano de Neurorrehabilitación (FIVAN), 46005, Valencia, Spain

^58^ Department of Neurosurgery, Shanghai Renji Hospital, School of Medicine, Shanghai Jiaotong University, 200127, Shanghai, China

^59^ Karolinska Institutet, INCF International Neuroinformatics Coordinating Facility, 17176, Stockholm, Sweden

^60^ Emergency Department, CHU, 4000, Liège, Belgium

^61^ Neurosurgery clinic, Pauls Stradins Clinical University Hospital, 1002, Riga, Latvia

^62^ Department of Computing, Imperial College London, SW7 2BU, London, UK

^63^ Department of Neurosurgery, Hospital Universitario 12 de Octubre, 28041 Madrid, Spain

^64^ Department of Anesthesia, Critical Care and Pain Medicine, Medical University of Vienna, 1090, Vienna, Austria

^65^ College of Health and Medicine, Australian National University, 2601, Canberra, Australia

^66^ Department of Neurosurgery, Neurosciences Centre & JPN Apex trauma centre, All India Institute of Medical Sciences, 110029, New Delhi, India

^67^ Department of Neurosurgery, Erasmus MC, 3015 CE, Rotterdam, the Netherlands

^68^ Department of Neurosurgery, Oslo University Hospital, 0424, Oslo, Norway

^69^ Division of Psychology, University of Stirling, FK9 4LA, Stirling, UK

^70^ Division of Neurosurgery, Department of Clinical Neurosciences, Addenbrooke’s Hospital & University of Cambridge, CB2 0QQ, Cambridge, UK

^71^ Department of Neurology, University of Groningen, University Medical Center Groningen, 9700 RB, Groningen, Netherlands

^72^ Neurointensive Care, Sheffield Teaching Hospitals NHS Foundation Trust, S10 2JF, Sheffield, UK

^73^ Salford Royal Hospital NHS Foundation Trust Acute Research Delivery Team, M5 5AP, Salford, UK

^74^ Department of Intensive Care and Department of Ethics and Philosophy of Medicine, Erasmus Medical Center, 3015 CE, Rotterdam, The Netherlands

^75^ Department of Clinical Neuroscience, Neurosurgery, Umeå University, 90185, Umeå, Sweden

^76^ Hungarian Brain Research Program—Grant No. KTIA_13_NAP-A-II/8, University of Pécs, 7622, Pécs, Hungary

^77^ Cyclotron Research Center, University of Liège, 4000, Liège, Belgium

^78^ Centre for Urgent and Emergency Care Research (CURE), Health Services Research Section, School of Health and Related Research (ScHARR), University of Sheffield, S10 2TG, Sheffield, UK

^79^ Emergency Department, Salford Royal Hospital, M6 (HD, Salford, UK

^80^ Institute of Research in Operative Medicine (IFOM), Witten/Herdecke University, 58455, Cologne, Germany

^81^ VP Global Project Management CNS, ICON, 92000, Paris, France

^82^ Department of Anesthesiology-Intensive Care, Lille University Hospital, 59037, Lille, France

^83^ Department of Neurosurgery, Rambam Medical Center, 31096, Haifa, Israel

^84^ Department of Anesthesiology & Intensive Care, University Hospitals Southhampton NHS Trust, SO16 6YD, Southhampton, UK

^85^ Cologne-Merheim Medical Center (CMMC), Department of Traumatology, Orthopedic Surgery and Sportmedicine, Witten/Herdecke University, 58445, Cologne, Germany

^86^ Intensive Care Unit, Southmead Hospital, Bristol, BS10 5NB, Bristol, UK

^87^ Department of Neurological Surgery, University of California, CA 94110, San Francisco, CA, USA

^88^ Department of Anesthesia & Intensive Care, M. Bufalini Hospital, 47521, Cesena, Italy

^89^ Department of Neurosurgery, University Hospital Heidelberg, 69120, Heidelberg, Germany

^90^ Department of Neurosurgery, The Walton centre NHS Foundation Trust, L9 7LI, Liverpool, UK

^91^ Department of Medical Genetics, University of Pécs, 7622, Pécs, Hungary

^92^ Department of Neurosurgery, Emergency County Hospital Timisoara, 300736, Timisoara, Romania

^93^ School of Medical Sciences, Örebro University, 70281, Örebro, Sweden

^94^ Institute for Molecular Medicine Finland, University of Helsinki, 00290, Helsinki, Finland

^95^ Analytic and Translational Genetics Unit, Department of Medicine; Psychiatric & Neurodevelopmental Genetics Unit, Department of Psychiatry; Department of Neurology, Massachusetts General Hospital, MA 02114, Boston, MA, USA

^96^ Program in Medical and Population Genetics; The Stanley Center for Psychiatric Research, The Broad Institute of MIT and Harvard, MA 02142, Cambridge, MA, USA

^97^ Department of Radiology, University of Antwerp, 2650, Edegem, Belgium

^98^ Department of Anesthesiology & Intensive Care, University Hospital of Grenoble, 38043, Grenoble, France

^99^ Department of Anesthesia & Intensive Care, Azienda Ospedaliera Università di Padova, 35100, Padova, Italy

^100^ Department of Neurosurgery, Leiden University Medical Center, 2300 RC, Leiden, The Netherlands and Department of Neurosurgery, Medical Center Haaglanden, 2597 AX, The Hague, The Netherlands

^101^ Department of Neurosurgery, Helsinki University Central Hospital, 00290, Helsinki, Finland

^102^ Division of Clinical Neurosciences, Department of Neurosurgery and Turku Brain Injury Centre, Turku University Hospital and University of Turku, 20521, Turku, Finland

^103^ Department of Anesthesiology and Critical Care, Pitié -Salpêtrière Teaching Hospital, Assistance Publique, Hôpitaux de Paris and University Pierre et Marie Curie, 75013, Paris, France

^104^ Neurotraumatology and Neurosurgery Research Unit (UNINN), Vall d’Hebron Research Institute, 08035, Barcelona, Spain

^105^ Department of Neurosurgery, Kaunas University of technology and Vilnius University, 44249, Vilnius, Lithuania

^106^ Department of Neurosurgery, Rezekne Hospital, 4604, Rezenke, Latvia

^107^ Department of Anesthesia, Critical Care & Pain Medicine NHS Lothian & University of Edinburg, EH4 2XU, Edinburgh, UK

^108^ Director, MRC Biostatistics Unit, Cambridge Institute of Public Health, Cb2 0SR, Cambridge, UK

^109^ Department of Physical Medicine and Rehabilitation, Oslo University Hospital, University of Oslo, 0424, Oslo, Norway

^110^ Division of Orthopedics, Oslo University Hospital, 0424, Oslo, Norway

^111^ Institute of Clinical Medicine, Faculty of Medicine, University of Oslo, 0424, Oslo, Norway

^112^ Broad Institute, Cambridge MA Harvard Medical School, Boston MA, Massachusetts General Hospital, MA 02114, Boston, MA, USA

^113^ National Trauma Research Institute, The Alfred Hospital, Monash University, 3004, Melbourne, Victoria, Australia

^114^ Department of Neurosurgery, Odense University Hospital, 5000, Odense, Denmark

^115^ International Neurotrauma Research Organization, 1090, Vienna, Austria

^116^ Klinik für Neurochirurgie, Klinikum Ludwigsburg, 71640, Ludwigsburg, Germany

^117^ Division of Biostatistics and Epidemiology, Department of Preventive Medicine, University of Debrecen, 4032, Debrecen, Hungary

^118^ Department Health and Prevention, University Greifswald, 17489, Greifswald, Germany

^119^ Department of Anesthesiology and Intensive Care, AUVA Trauma Hospital, 5026, Salzburg, Austria

^120^ Department of Neurology, Elisabeth-TweeSteden Ziekenhuis, 5022 GC, Tilburg, the Netherlands

^121^ Department of Neuroanesthesia and Neurointensive Care, Odense University Hospital, 5000, Odense, Denmark

^122^ Department of Neuromedicine and Movement Science, Norwegian University of Science and Technology, NTNU, 7491, Trondheim, Norway

^123^ Department of Physical Medicine and Rehabilitation, St. Olavs Hospital, Trondheim University Hospital, 7491, Trondheim, Norway

^124^ Department of Neurosurgery, University of Pécs, 7622, Pécs, Hungary

^125^ Division of Neuroscience Critical Care, John Hopkins University School of Medicine, MD 21205, Baltimore, MD, USA

^126^ Department of Neuropathology, Queen Elizabeth University Hospital and University of Glasgow, G51 4TF, Glasgow, UK

^127^ Department of Department of Biomedical Data Sciences, Leiden University Medical Center, 2300 RC, Leiden, The Netherlands

^128^ Department of Pathophysiology and Transplantation, Milan University, and Neuroscience ICU, Fondazione IRCCS Cà Granda Ospedale Maggiore Policlinico, 20122, Milano, Italy

^129^ Department of Radiation Sciences, Biomedical Engineering, Umeå University, 90185, Umeå, Sweden

^130^ Cochrane Consumers and Communication Review Group, Centre for Health Communication and Participation, School of Psychology and Public Health, La Trobe University, 3004, Melbourne, Australia

^131^ Perioperative Services, Intensive Care Medicine and Pain Management, Turku University Hospital and University of Turku, 20521, Turku, Finland

^132^ Department of Neurosurgery, Kaunas University of Health Sciences, 50009, Kaunas, Lithuania

^133^ Intensive Care and Department of Pediatric Surgery, Erasmus Medical Center, Sophia Children’s Hospital, 3015 CE, Rotterdam, The Netherlands

^134^ Department of Neurosurgery, Kings college London, WC2R 2LS, London, UK

^135^ Neurologie, Neurochirurgie und Psychiatrie, Charité—Universitätsmedizin Berlin, 13353, Berlin, Germany

^136^ Department of Intensive Care Adults, Erasmus MC—University Medical Center Rotterdam, 30115 CE, Rotterdam, The Netherlands

^137^ icoMetrix NV, 3000, Leuven, Belgium

^138^ Movement Science Group, Faculty of Health and Life Sciences, Oxford Brookes University, OX3 0BP, Oxford, UK

^139^ Psychology Department, Antwerp University Hospital, 2650, Edegem, Belgium

^140^ Director of Neurocritical Care, University of California, CA 90095, Los Angeles, CA, USA

^141^ Department of Neurosurgery, St.Olavs Hospital, Trondheim University Hospital, 7491, Trondheim, Norway

^142^ Department of Emergency Medicine, University of Florida, Gainesville, FL 32611, FL, USA

^143^ Department of Neurosurgery, Charité—Universitätsmedizin Berlin, corporate member of Freie Universität Berlin, Humboldt-Universität zu Berlin, and Berlin Institute of Health, 13353, Berlin, Germany

^144^ VTT Technical Research Centre, 02044, Tampere, Finland

^145^ Section of Neurosurgery, Department of Surgery, Rady Faculty of Health Sciences, University of Manitoba, R3E 3P5, Winnipeg, MB, Canada
