# Supplementary material for: Reference Values of the QOLIBRI from General Population Samples in the United Kingdom and The Netherlands
Source: J Clin Med. 2020 Jul 3;9(7):2100. doi: 10.3390/jcm9072100 (PMC7408671; doi:10.3390/jcm9072100)
Supplement: Supplementary file 1 [file jcm-09-02100-s001.zip › OS1_QOLIBRI_UK.pdf]

**Sex x Health status x Age**				**Low HRQoL**		**-1 *SD***			***Md***			**+1 *SD***		**High HRQoL**
Sex	Health status	Age	**N**	**2.5%**	**5%**	**16%**	**30%**	**40%**	**50%**	**60%**	**70%**	**85%**	**95%**	**97.25%**
Female	Healthy	Age: 18-40	434	32	40	50	68	75	75	83	90	100	100	100
		Age: 41-64	408	47	50	68	75	83	86	93	97	100	100	100
		Age: 65-75	119	54	64	75	90	93	97	97	100	100	100	100
	At least one chronic condition	Age: 18-40	547	15	22	36	47	50	61	68	75	86	100	100
		Age: 41-64	587	8	15	40	50	58	68	75	83	93	100	100
		Age: 65-75	174	27	40	57	75	79	86	90	93	100	100	100
Male	Healthy	Age: 18-40	497	29	36	50	65	72	75	83	86	97	100	100
		Age: 41-64	442	43	50	70	75	79	86	93	97	100	100	100
		Age: 65-75	116	43	53	75	83	90	93	97	97	100	100	100
	At least one chronic condition	Age: 18-40	407	11	22	36	47	50	58	65	72	83	97	100
		Age: 41-64	517	11	18	43	50	61	72	78	86	97	100	100
		Age: 65-75	155	28	36	61	72	75	83	90	93	100	100	100
**Sex x Health status x Education**				**Low HRQoL**		**-1 *SD***			***Md***			**+1 *SD***		**High HRQoL**
Sex	Health status	Education	**N**	**2.5%**	**5%**	**16%**	**30%**	**40%**	**50%**	**60%**	**70%**	**85%**	**95%**	**97.25%**
Female	Healthy	education: low	193	33	43	50	72	75	86	90	97	100	100	100
		education: middle	383	40	50	65	75	79	86	90	97	100	100	100
		education: high	385	40	50	61	75	75	83	90	93	100	100	100
	At least one chronic condition	education: low	332	15	24	36	50	56	68	75	83	97	100	100
		education: middle	526	8	18	36	50	58	68	75	79	93	100	100
		education: high	450	15	22	40	50	61	72	75	83	93	100	100
Male	Healthy	education: low	197	25	25	50	72	75	79	86	93	100	100	100
		education: middle	493	41	47	58	72	75	79	86	93	100	100	100
		education: high	365	36	43	61	75	79	86	90	93	100	100	100
	At least one chronic condition	education: low	280	11	15	36	47	50	61	72	75	93	100	100
		education: middle	482	11	22	40	50	61	68	75	83	97	100	100
		education: high	317	22	29	43	57	65	72	75	83	97	100	100
		Total	4403	18	25	47	61	72	75	83	90	97	100	100

Note. HRQoL: health-related quality of life; 50% percentiles represent 50% of the distribution corresponding to the median (Md); SD: standard deviation; values from −1 standard deviation (16%) to +1 standard deviation (85%) are within the normal range (i.e., not impaired HRQoL); values below 16% indicate impaired HRQoL and values above 85% indicate outstanding HRQoL.

Sex x Health status x Age				Low HRQoL		-1 *SD*			*Md*			+1 *SD*		High HRQoL
Sex	Health status	Age	**N**	**2.5%**	**5%**	**16%**	**30%**	**40%**	**50%**	**60%**	**70%**	**85%**	**95%**	**97.25%**
Female	Healthy	Age: 18-40	434	14	21	36	50	54	61	68	75	86	100	100
		Age: 41-64	408	18	25	43	54	65	72	75	79	93	100	100
		Age: 65-75	119	36	40	54	63	72	75	79	86	94	100	100
	At least one chronic condition	Age: 18-40	547	0	0	8	18	25	36	43	50	61	79	91
		Age: 41-64	587	0	0	11	25	33	43	49	54	72	86	95
		Age: 65-75	174	11	17	32	47	54	61	65	72	83	95	97
Male	Healthy	Age: 18-40	497	25	29	43	50	58	65	72	75	86	100	100
		Age: 41-64	442	22	33	50	61	68	75	75	83	97	100	100
		Age: 65-75	116	39	50	61	72	75	75	79	86	93	100	100
	At least one chronic condition	Age: 18-40	407	0	4	15	29	36	43	50	54	68	79	93
		Age: 41-64	517	0	4	15	33	40	50	54	61	75	93	97
		Age: 65-75	155	7	24	38	54	61	65	72	75	86	93	98
**Sex x Health status x Education**				**Low HRQoL**		**-1 *SD***			***Md***			**+1 *SD***		**High HRQoL**
Sex	Health status	Education	**N**	**2.5%**	**5%**	**16%**	**30%**	**40%**	**50%**	**60%**	**70%**	**85%**	**95%**	**97.25%**
Female	Healthy	education: low	193	18	25	43	50	61	68	75	79	93	100	100
		education: middle	383	18	25	43	50	58	68	75	75	92	100	100
		education: high	385	11	25	43	54	61	68	73	79	86	100	100
	At least one chronic condition	education: low	332	0	0	15	25	36	43	50	58	72	93	100
		education: middle	526	0	0	15	25	33	40	50	54	69	86	93
		education: high	450	0	0	11	25	36	43	48	58	72	86	93
Male	Healthy	education: low	197	22	33	50	58	68	75	79	86	97	100	100
		education: middle	493	25	29	47	54	61	68	75	79	90	100	100
		education: high	365	25	36	50	58	65	72	75	79	90	100	100
	At least one chronic condition	education: low	280	0	4	18	33	40	47	54	61	75	93	97
		education: middle	482	0	4	15	29	40	47	50	61	72	90	97
		education: high	317	4	8	25	40	47	50	61	68	75	93	97
		Total	4403	0	8	25	43	50	54	65	72	83	100	100

Note. HRQoL: health-related quality of life; 50% percentiles represent 50% of the distribution corresponding to the median (Md); SD: standard deviation; values from −1 standard deviation (16%) to +1 standard deviation (85%) are within the normal range (i.e., not impaired HRQoL); values below 16% indicate impaired HRQoL and values above 85% indicate outstanding HRQoL.

**Sex x Health status x Age**				**Low HRQoL**		**-1 *SD***			***Md***			**+1 *SD***		**High HRQoL**
Sex	Health status	Age	**N**	**2.5%**	**5%**	**16%**	**30%**	**40%**	**50%**	**60%**	**70%**	**85%**	**95%**	**97.25%**
Female	Healthy	Age: 18-40	434	33	40	54	68	72	75	82	87	97	100	100
		Age: 41-64	408	44	50	65	75	83	86	93	97	100	100	100
		Age: 65-75	119	50	54	75	83	86	90	97	97	100	100	100
	At least one chronic condition	Age: 18-40	547	4	8	25	40	47	50	61	68	83	93	100
		Age: 41-64	587	4	8	25	40	50	58	68	75	86	99	100
		Age: 65-75	174	16	25	46	58	65	72	79	86	93	100	100
Male	Healthy	Age: 18-40	497	36	40	50	61	68	75	79	86	93	100	100
		Age: 41-64	442	47	50	65	75	79	86	90	97	100	100	100
		Age: 65-75	116	54	65	75	83	86	90	93	97	100	100	100
	At least one chronic condition	Age: 18-40	407	8	11	29	40	47	50	58	65	75	90	93
		Age: 41-64	517	0	8	29	43	50	58	68	75	92	100	100
		Age: 65-75	155	19	28	50	58	68	75	83	86	97	100	100
**Sex x Health status x Education**				**Low HRQoL**		**-1 *SD***			***Md***			**+1 *SD***		**High HRQoL**
Sex	Health status	Education	**N**	**2.5%**	**5%**	**16%**	**30%**	**40%**	**50%**	**60%**	**70%**	**85%**	**95%**	**97.25%**
Female	Healthy	education: low	193	36	47	54	70	75	83	86	93	100	100	100
		education: middle	383	43	50	61	72	75	83	90	93	100	100	100
		education: high	385	40	44	61	72	75	83	86	93	100	100	100
	At least one chronic condition	education: low	332	8	15	25	36	47	50	61	68	83	99	100
		education: middle	526	0	8	25	43	50	58	65	72	86	97	100
		education: high	450	4	9	32	47	53	63	72	75	90	100	100
Male	Healthy	education: low	197	36	46	52	68	75	79	86	90	100	100	100
		education: middle	493	40	45	54	68	75	79	86	90	100	100	100
		education: high	365	43	48	61	75	79	83	90	93	100	100	100
	At least one chronic condition	education: low	280	0	8	25	40	47	50	58	68	86	100	100
		education: middle	482	4	11	29	43	50	58	65	75	90	100	100
		education: high	317	11	18	40	50	56	61	68	75	90	100	100
		Total	4403	8	18	40	54	61	72	75	86	97	100	100

Note. HRQoL: health-related quality of life; 50% percentiles represent 50% of the distribution corresponding to the median (Md); SD: standard deviation; values from −1 standard deviation (16%) to +1 standard deviation (85%) are within the normal range (i.e., not impaired HRQoL); values below 16% indicate impaired HRQoL and values above 85% indicate outstanding HRQoL.

**Sex x Health status x Age**				**Low HRQoL**		**-1 *SD***			***Md***			**+1 *SD***		**High HRQoL**
Sex	Health status	Age	**N**	**2.5%**	**5%**	**16%**	**30%**	**40%**	**50%**	**60%**	**70%**	**85%**	**95%**	**97.25%**
Female	Healthy	Age: 18-40	434	25	34	50	63	71	75	75	84	96	100	100
		Age: 41-64	408	34	39	50	67	75	80	88	92	100	100	100
		Age: 65-75	119	42	50	58	71	75	88	92	96	100	100	100
	At least one chronic condition	Age: 18-40	547	0	9	25	38	46	50	59	67	80	92	100
		Age: 41-64	587	5	13	30	46	50	59	67	75	88	100	100
		Age: 65-75	174	25	32	46	63	67	75	83	88	96	100	100
Male	Healthy	Age: 18-40	497	25	30	46	50	59	67	71	75	92	100	100
		Age: 41-64	442	25	34	50	63	71	75	84	88	100	100	100
		Age: 65-75	116	49	54	67	75	75	80	88	92	99	100	100
	At least one chronic condition	Age: 18-40	407	0	9	25	38	46	50	55	63	75	92	100
		Age: 41-64	517	0	9	30	42	50	59	67	75	88	100	100
		Age: 65-75	155	17	28	49	59	67	75	80	84	96	100	100
**Sex x Health status x Education**				**Low HRQoL**		**-1 *SD***			***Md***			**+1 *SD***		**High HRQoL**
Sex	Health status	Education	**N**	**2.5%**	**5%**	**16%**	**30%**	**40%**	**50%**	**60%**	**70%**	**85%**	**95%**	**97.25%**
Female	Healthy	education: low	193	30	38	50	63	71	75	84	92	100	100	100
		education: middle	383	25	38	50	63	71	75	84	92	100	100	100
		education: high	385	30	42	55	67	71	75	80	91	100	100	100
	At least one chronic condition	education: low	332	10	17	30	46	55	59	67	75	92	100	100
		education: middle	526	0	9	25	42	50	55	67	75	88	100	100
		education: high	450	5	13	30	42	50	59	67	75	88	96	100
Male	Healthy	education: low	197	25	38	50	59	67	75	78	88	96	100	100
		education: middle	493	25	30	46	55	67	71	75	84	96	100	100
		education: high	365	30	38	50	63	71	75	80	88	96	100	100
	At least one chronic condition	education: low	280	0	13	30	42	50	55	63	71	84	100	100
		education: middle	482	0	9	25	42	50	55	63	75	84	100	100
		education: high	317	5	13	34	46	50	59	67	75	88	100	100
		Total	4403	9	17	38	50	59	67	75	80	92	100	100

Note. HRQoL: health-related quality of life; 50% percentiles represent 50% of the distribution corresponding to the median (Md); SD: standard deviation; values from −1 standard deviation (16%) to +1 standard deviation (85%) are within the normal range (i.e., not impaired HRQoL); values below 16% indicate impaired HRQoL and values above 85% indicate outstanding HRQoL.

**Sex x Health status x Age**				**Low HRQoL**		**-1 *SD***			***Md***			**+1 *SD***		**High HRQoL**
Sex	Health status	Age	**N**	**2.5%**	**5%**	**16%**	**30%**	**40%**	**50%**	**60%**	**70%**	**85%**	**95%**	**97.25%**
Female	Healthy	Age: 18-40	434	16	20	31	45	50	56	60	70	86	100	100
		Age: 41-64	408	25	31	50	65	75	80	86	95	100	100	100
		Age: 65-75	119	35	49	60	80	86	90	95	100	100	100	100
	At least one chronic condition	Age: 18-40	547	0	5	20	31	35	40	50	56	70	80	87
		Age: 41-64	587	9	16	31	40	50	56	65	75	86	100	100
		Age: 65-75	174	17	25	50	60	70	80	86	90	100	100	100
Male	Healthy	Age: 18-40	497	10	20	35	45	50	56	65	71	90	100	100
		Age: 41-64	442	25	31	50	60	70	80	86	95	100	100	100
		Age: 65-75	116	30	35	63	80	86	90	95	98	100	100	100
	At least one chronic condition	Age: 18-40	407	0	5	25	35	40	45	50	56	70	86	95
		Age: 41-64	517	0	10	31	45	50	60	65	75	90	100	100
		Age: 65-75	155	20	29	49	61	70	80	90	95	100	100	100
**Sex x Health status x Education**				**Low HRQoL**		**-1 *SD***			***Md***			**+1 *SD***		**High HRQoL**
Sex	Health status	Education	**N**	**2.5%**	**5%**	**16%**	**30%**	**40%**	**50%**	**60%**	**70%**	**85%**	**95%**	**97.25%**
Female	Healthy	education: low	193	18	29	45	50	60	70	80	86	100	100	100
		education: middle	383	20	25	40	53	65	75	86	90	100	100	100
		education: high	385	20	22	40	50	60	70	75	80	95	100	100
	At least one chronic condition	education: low	332	5	10	25	40	50	56	60	70	90	100	100
		education: middle	526	0	10	25	35	45	50	60	70	80	95	100
		education: high	450	5	10	25	35	45	50	60	65	80	95	100
Male	Healthy	education: low	197	20	25	40	50	60	70	80	90	100	100	100
		education: middle	493	20	25	40	50	60	70	75	86	100	100	100
		education: high	365	16	25	40	50	60	70	80	90	100	100	100
	At least one chronic condition	education: low	280	0	10	29	40	50	56	65	72	91	100	100
		education: middle	482	0	5	25	40	45	56	60	70	90	100	100
		education: high	317	10	16	31	45	50	56	65	70	88	100	100
		Total	4403	5	16	31	45	50	60	70	80	95	100	100

Note. HRQoL: health-related quality of life; 50% percentiles represent 50% of the distribution corresponding to the median (Md); SD: standard deviation; values from −1 standard deviation (16%) to +1 standard deviation (85%) are within the normal range (i.e., not impaired HRQoL); values below 16% indicate impaired HRQoL and values above 85% indicate outstanding HRQoL.

**Sex x Health status x Age**				**Low HRQoL**		**-1 *SD***			***Md***			**+1 *SD***		**High HRQoL**
Sex	Health status	Age	**N**	**2.5%**	**5%**	**16%**	**30%**	**40%**	**50%**	**60%**	**70%**	**85%**	**95%**	**97.25%**
Female	Healthy	Age: 18-40	434	25	31	50	65	75	86	90	95	100	100	100
		Age: 41-64	408	35	40	60	75	86	90	95	100	100	100	100
		Age: 65-75	119	35	40	56	75	86	90	95	95	100	100	100
	At least one chronic condition	Age: 18-40	547	9	20	35	50	56	65	70	80	90	100	100
		Age: 41-64	587	5	10	31	45	50	60	65	75	86	95	100
		Age: 65-75	174	19	35	40	50	60	65	70	80	90	95	100
Male	Healthy	Age: 18-40	497	22	31	45	50	65	75	86	95	100	100	100
		Age: 41-64	442	35	40	56	75	80	86	93	100	100	100	100
		Age: 65-75	116	35	49	75	86	86	90	95	98	100	100	100
	At least one chronic condition	Age: 18-40	407	11	20	35	45	50	56	60	70	86	95	100
		Age: 41-64	517	5	16	35	49	50	60	70	75	90	100	100
		Age: 65-75	155	16	20	40	50	59	70	75	80	90	100	100
**Sex x Health status x Education**				**Low HRQoL**		**-1 *SD***			***Md***			**+1 *SD***		**High HRQoL**
Sex	Health status	Education	**N**	**2.5%**	**5%**	**16%**	**30%**	**40%**	**50%**	**60%**	**70%**	**85%**	**95%**	**97.25%**
Female	Healthy	education: low	193	31	35	50	60	75	80	90	95	100	100	100
		education: middle	383	31	40	56	75	80	90	95	95	100	100	100
		education: high	385	25	35	50	75	80	90	90	95	100	100	100
	At least one chronic condition	education: low	332	2	13	35	45	50	60	65	75	86	100	100
		education: middle	526	1	10	35	50	50	60	70	75	86	95	100
		education: high	450	16	25	40	50	60	65	70	80	90	100	100
Male	Healthy	education: low	197	25	31	50	60	75	86	90	96	100	100	100
		education: middle	493	25	35	50	65	75	86	90	95	100	100	100
		education: high	365	31	35	50	65	75	86	90	95	100	100	100
	At least one chronic condition	education: low	280	10	16	35	45	50	56	65	70	86	96	100
		education: middle	482	10	20	35	45	56	60	70	75	90	100	100
		education: high	317	15	25	40	50	50	60	70	80	90	100	100
		Total	4403	16	25	40	50	60	70	80	90	100	100	100

Note. HRQoL: health-related quality of life; 50% percentiles represent 50% of the distribution corresponding to the median (Md); SD: standard deviation; values from −1 standard deviation (16%) to +1 standard deviation (85%) are within the normal range (i.e., not impaired HRQoL); values below 16% indicate impaired HRQoL and values above 85% indicate outstanding HRQoL.
