# Supplementary material for: Reference Values of the QOLIBRI from General Population Samples in the United Kingdom and The Netherlands
Source: J Clin Med. 2020 Jul 3;9(7):2100. doi: 10.3390/jcm9072100 (PMC7408671; doi:10.3390/jcm9072100)
Supplement: Supplementary file 1 [file jcm-09-02100-s001.zip › OS2_QOLIBRI_NL.pdf]

**Sex x Health status x Age**				**Low HRQoL**		**-1 *SD***			***Md***			**+1 *SD***		**High HRQoL**
Sex	Health status	Age	**N**	**2.5%**	**5%**	**16%**	**30%**	**40%**	**50%**	**60%**	**70%**	**85%**	**95%**	**97.25%**
Female	Healthy	Age: 18-40	338	47	50	68	75	75	83	86	90	100	100	100
		Age: 41-64	292	58	63	75	75	83	86	93	97	100	100	100
		Age: 65-75	66	66	72	75	75	83	86	90	97	100	100	100
	At least one chronic condition	Age: 18-40	364	29	36	50	61	68	74	75	83	90	100	100
		Age: 41-64	527	36	50	61	72	75	79	83	90	97	100	100
		Age: 65-75	147	39	54	68	75	79	83	90	93	100	100	100
Male	Healthy	Age: 18-40	388	43	50	61	75	75	83	86	93	100	100	100
		Age: 41-64	396	50	58	75	75	83	90	97	97	100	100	100
		Age: 65-75	92	73	75	75	79	90	93	97	100	100	100	100
	At least one chronic condition	Age: 18-40	248	25	30	47	54	58	63	72	75	86	100	100
		Age: 41-64	436	29	40	58	72	75	75	83	90	97	100	100
		Age: 65-75	105	37	51	72	75	79	90	93	97	100	100	100
**Sex x Health status x Education**				**Low HRQoL**		**-1 *SD***			***Md***			**+1 *SD***		**High HRQoL**
Sex	Health status	Education	**N**	**2.5%**	**5%**	**16%**	**30%**	**40%**	**50%**	**60%**	**70%**	**85%**	**95%**	**97.25%**
Female	Healthy	education: low	171	48	52	65	75	75	79	86	93	100	100	100
		education: middle	341	50	61	72	75	79	86	90	93	100	100	100
		education: high	184	55	62	75	79	83	86	93	97	100	100	100
	At least one chronic condition	education: low	374	33	40	54	68	72	75	79	86	97	100	100
		education: middle	477	29	40	58	72	75	75	83	86	97	100	100
		education: high	187	39	50	61	72	75	83	86	90	100	100	100
Male	Healthy	education: low	202	43	50	62	75	75	79	86	96	100	100	100
		education: middle	394	50	54	72	75	79	86	93	97	100	100	100
		education: high	280	50	54	75	79	83	90	93	97	100	100	100
	At least one chronic condition	education: low	277	25	33	50	68	72	75	75	83	93	100	100
		education: middle	314	25	36	54	65	72	75	83	86	97	100	100
		education: high	198	36	40	54	65	72	75	79	89	99	100	100
		Total	3399	36	47	61	72	75	79	86	90	100	100	100

Note. HRQoL: health-related quality of life; 50% percentiles represent 50% of the distribution corresponding to the median (Md); SD: standard deviation; values from −1 standard deviation (16%) to +1 standard deviation (85%) are within the normal range (i.e., not impaired HRQoL); values below 16% indicate impaired HRQoL and values above 85% indicate outstanding HRQoL.

**Sex x Health status x Age**				**Low HRQoL**		**-1 *SD***			***Md***			**+1 *SD***		**High HRQoL**
Sex	Health status	Age	**N**	**2.5%**	**5%**	**16%**	**30%**	**40%**	**50%**	**60%**	**70%**	**85%**	**95%**	**97.25%**
Female	Healthy	Age: 18-40	338	20	40	54	65	72	75	75	83	90	97	100
		Age: 41-64	292	33	42	61	72	75	75	79	83	97	100	100
		Age: 65-75	66	58	62	68	74	75	75	83	86	94	100	100
	At least one chronic condition	Age: 18-40	364	8	15	33	47	50	58	65	68	75	86	100
		Age: 41-64	527	18	25	43	54	61	68	72	75	86	93	97
		Age: 65-75	147	36	44	58	68	72	72	78	79	86	96	98
Male	Healthy	Age: 18-40	388	39	47	54	68	72	75	79	83	97	100	100
		Age: 41-64	396	43	50	65	75	75	79	86	90	97	100	100
		Age: 65-75	92	62	68	75	75	77	81	86	90	98	100	100
	At least one chronic condition	Age: 18-40	248	11	22	43	50	54	58	65	68	75	89	93
		Age: 41-64	436	15	25	43	54	61	68	72	75	83	94	100
		Age: 65-75	105	40	48	61	69	74	75	79	83	90	97	98
**Sex x Health status x Education**				**Low HRQoL**		**-1 *SD***			***Md***			**+1 *SD***		**High HRQoL**
Sex	Health status	Education	**N**	**2.5%**	**5%**	**16%**	**30%**	**40%**	**50%**	**60%**	**70%**	**85%**	**95%**	**97.25%**
Female	Healthy	education: low	171	16	33	54	68	75	75	75	79	90	100	100
		education: middle	341	40	47	61	68	72	75	79	86	93	100	100
		education: high	184	35	47	61	68	75	75	79	83	92	97	100
	At least one chronic condition	education: low	374	11	22	40	50	61	68	72	75	83	93	97
		education: middle	477	11	22	40	50	61	65	68	75	83	93	97
		education: high	187	25	27	43	54	58	68	72	75	83	93	100
Male	Healthy	education: low	202	43	50	61	72	75	75	83	89	97	100	100
		education: middle	394	43	50	61	72	75	79	83	86	97	100	100
		education: high	280	40	47	65	72	75	79	83	90	97	100	100
	At least one chronic condition	education: low	277	11	22	43	54	61	68	72	75	83	93	97
		education: middle	314	18	25	43	50	58	65	68	75	83	95	100
		education: high	198	22	33	47	54	61	68	72	75	86	93	100
		Total	3399	22	29	50	61	68	72	75	79	90	100	100

Note. HRQoL: health-related quality of life; 50% percentiles represent 50% of the distribution corresponding to the median (Md); SD: standard deviation; values from −1 standard deviation (16%) to +1 standard deviation (85%) are within the normal range (i.e., not impaired HRQoL); values below 16% indicate impaired HRQoL and values above 85% indicate outstanding HRQoL.

**Sex x Health status x Age**				**Low HRQoL**		**-1 *SD***			***Md***			**+1 *SD***		**High HRQoL**
Sex	Health status	Age	**N**	**2.5%**	**5%**	**16%**	**30%**	**40%**	**50%**	**60%**	**70%**	**85%**	**95%**	**97.25%**
Female	Healthy	Age: 18-40	338	50	50	65	75	75	83	90	93	100	100	100
		Age: 41-64	292	50	58	75	75	83	86	93	97	100	100	100
		Age: 65-75	66	67	68	75	79	86	91	97	97	100	100	100
	At least one chronic condition	Age: 18-40	364	18	29	47	58	65	72	75	79	90	97	100
		Age: 41-64	527	29	36	50	65	68	75	79	83	93	100	100
		Age: 65-75	147	46	50	61	72	75	83	86	90	97	100	100
Male	Healthy	Age: 18-40	388	43	50	61	72	75	79	86	90	100	100	100
		Age: 41-64	396	50	57	72	75	83	86	93	97	100	100	100
		Age: 65-75	92	65	70	75	79	86	93	97	100	100	100	100
	At least one chronic condition	Age: 18-40	248	23	29	43	54	58	63	68	75	83	97	100
		Age: 41-64	436	18	29	50	59	68	72	75	79	92	100	100
		Age: 65-75	105	33	50	60	68	75	79	86	90	97	100	100
**Sex x Health status x Education**				**Low HRQoL**		**-1 *SD***			***Md***			**+1 *SD***		**High HRQoL**
Sex	Health status	Education	**N**	**2.5%**	**5%**	**16%**	**30%**	**40%**	**50%**	**60%**	**70%**	**85%**	**95%**	**97.25%**
Female	Healthy	education: low	171	48	50	65	75	75	79	86	93	97	100	100
		education: middle	341	50	54	72	75	79	86	90	97	100	100	100
		education: high	184	54	62	72	75	83	90	93	97	100	100	100
	At least one chronic condition	education: low	374	25	33	50	61	68	72	75	83	90	100	100
		education: middle	477	29	33	50	61	68	75	78	83	93	100	100
		education: high	187	31	40	54	68	72	75	83	86	93	100	100
Male	Healthy	education: low	202	47	50	65	75	75	79	86	93	100	100	100
		education: middle	394	50	50	68	75	79	83	90	97	100	100	100
		education: high	280	43	50	68	75	83	90	93	97	100	100	100
	At least one chronic condition	education: low	277	18	25	47	54	61	68	72	75	90	97	100
		education: middle	314	25	33	50	58	65	72	75	83	93	100	100
		education: high	198	32	39	52	61	71	75	76	83	93	100	100
		Total	3399	29	40	54	68	75	75	83	90	97	100	100

Note. HRQoL: health-related quality of life; 50% percentiles represent 50% of the distribution corresponding to the median (Md); SD: standard deviation; values from −1 standard deviation (16%) to +1 standard deviation (85%) are within the normal range (i.e., not impaired HRQoL); values below 16% indicate impaired HRQoL and values above 85% indicate outstanding HRQoL.

**Sex x Health status x Age**				**Low HRQoL**		**-1 *SD***			***Md***			**+1 *SD***		**High HRQoL**
Sex	Health status	Age	**N**	**2.5%**	**5%**	**16%**	**30%**	**40%**	**50%**	**60%**	**70%**	**85%**	**95%**	**97.25%**
Female	Healthy	Age: 18-40	338	42	50	63	75	75	80	88	92	100	100	100
		Age: 41-64	292	42	49	67	75	75	82	88	96	100	100	100
		Age: 65-75	66	46	53	71	75	80	84	92	94	97	100	100
	At least one chronic condition	Age: 18-40	364	25	34	50	59	67	71	75	80	92	100	100
		Age: 41-64	527	30	38	50	67	71	75	80	88	92	100	100
		Age: 65-75	147	49	52	63	71	73	75	80	84	92	100	100
Male	Healthy	Age: 18-40	388	32	44	58	67	75	75	84	88	100	100	100
		Age: 41-64	396	42	50	63	73	75	80	84	92	96	100	100
		Age: 65-75	92	55	59	71	75	75	80	88	92	100	100	100
	At least one chronic condition	Age: 18-40	248	19	30	42	50	55	63	71	75	84	96	100
		Age: 41-64	436	13	21	46	59	63	71	75	80	88	100	100
		Age: 65-75	105	41	46	59	67	75	80	84	88	96	96	98
**Sex x Health status x Education**				**Low HRQoL**		**-1 *SD***			***Md***			**+1 *SD***		**High HRQoL**
Sex	Health status	Education	**N**	**2.5%**	**5%**	**16%**	**30%**	**40%**	**50%**	**60%**	**70%**	**85%**	**95%**	**97.25%**
Female	Healthy	education: low	171	38	42	59	75	75	80	84	92	100	100	100
		education: middle	341	46	50	67	75	80	84	88	92	100	100	100
		education: high	184	46	50	67	75	75	82	88	92	100	100	100
	At least one chronic condition	education: low	374	30	37	55	67	71	75	80	84	92	100	100
		education: middle	477	30	38	50	63	71	75	80	84	92	100	100
		education: high	187	25	35	55	67	71	75	80	84	92	100	100
Male	Healthy	education: low	202	38	42	55	67	75	75	82	88	96	100	100
		education: middle	394	38	49	63	71	75	80	84	92	100	100	100
		education: high	280	38	50	59	75	75	80	84	92	100	100	100
	At least one chronic condition	education: low	277	17	21	46	55	63	67	75	80	88	96	100
		education: middle	314	13	25	46	55	63	71	75	80	88	100	100
		education: high	198	25	33	50	59	66	71	75	80	88	97	100
		Total	3399	30	38	55	67	75	75	80	88	96	100	100

Note. HRQoL: health-related quality of life; 50% percentiles represent 50% of the distribution corresponding to the median (Md); SD: standard deviation; values from −1 standard deviation (16%) to +1 standard deviation (85%) are within the normal range (i.e., not impaired HRQoL); values below 16% indicate impaired HRQoL and values above 85% indicate outstanding HRQoL.

**Sex x Health status x Age**				**Low HRQoL**		***-1 SD***			***Md***			***+1 SD***		**High HRQoL**
Sex	Health status	Age	**N**	**2.5%**	**5%**	**16%**	**30%**	**40%**	**50%**	**60%**	**70%**	**85%**	**95%**	**97.25%**
Female	Healthy	Age: 18-40	338	25	25	45	56	65	73	80	86	95	100	100
		Age: 41-64	292	25	35	50	65	75	80	86	95	100	100	100
		Age: 65-75	66	24	47	62	75	86	90	90	100	100	100	100
	At least one chronic condition	Age: 18-40	364	16	20	35	45	50	56	60	70	80	90	100
		Age: 41-64	527	20	25	45	56	65	75	80	86	95	100	100
		Age: 65-75	147	29	35	56	65	73	75	83	90	95	100	100
Male	Healthy	Age: 18-40	388	16	25	40	56	60	70	75	86	95	100	100
		Age: 41-64	396	25	34	50	70	75	86	90	95	100	100	100
		Age: 65-75	92	47	56	75	80	86	90	95	100	100	100	100
	At least one chronic condition	Age: 18-40	248	16	20	31	40	45	50	50	60	75	90	100
		Age: 41-64	436	25	31	40	50	60	65	75	80	95	100	100
		Age: 65-75	105	40	40	56	75	75	86	90	90	100	100	100
**Sex x Health status x Education**				**Low HRQoL**		***-1 SD***			***Md***			***+1 SD***		**High HRQoL**
Sex	Health status	Education	**N**	**2.5%**	**5%**	**16%**	**30%**	**40%**	**50%**	**60%**	**70%**	**85%**	**95%**	**97.25%**
Female	Healthy	education: low	171	25	31	50	60	70	75	86	95	100	100	100
		education: middle	341	25	25	50	60	70	75	86	90	100	100	100
		education: high	184	25	35	50	60	70	80	84	90	100	100	100
	At least one chronic condition	education: low	374	16	25	40	50	60	70	75	86	95	100	100
		education: middle	477	20	25	40	56	60	65	75	80	90	100	100
		education: high	187	25	25	40	56	60	65	75	80	90	100	100
Male	Healthy	education: low	202	25	25	45	56	70	75	86	90	100	100	100
		education: middle	394	25	31	50	65	75	80	90	95	100	100	100
		education: high	280	20	31	50	60	75	80	86	92	100	100	100
	At least one chronic condition	education: low	277	20	25	40	50	56	65	73	80	90	100	100
		education: middle	314	20	25	35	45	50	60	65	76	90	100	100
		education: high	198	16	20	33	45	50	56	65	80	90	100	100
		Total	3399	20	25	40	56	65	70	80	86	100	100	100

Note. HRQoL: health-related quality of life; 50% percentiles represent 50% of the distribution corresponding to the median (Md); SD: standard deviation; values from −1 standard deviation (16%) to +1 standard deviation (85%) are within the normal range (i.e., not impaired HRQoL); values below 16% indicate impaired HRQoL and values above 85% indicate outstanding HRQoL.

**Sex x Health status x Age**				**Low HRQoL**		***-1 SD***			***Md***			***+1 SD***		**High HRQoL**
Sex	Health status	Age	**N**	**2.5%**	**5%**	**16%**	**30%**	**40%**	**50%**	**60%**	**70%**	**85%**	**95%**	**97.25%**
Female	Healthy	Age: 18-40	338	38	45	60	75	80	86	90	95	100	100	100
		Age: 41-64	292	42	50	65	75	80	86	90	95	100	100	100
		Age: 65-75	66	40	52	70	80	80	86	86	90	95	100	100
	At least one chronic condition	Age: 18-40	364	20	25	40	50	56	60	65	75	80	95	100
		Age: 41-64	527	16	25	35	45	56	60	70	75	86	99	100
		Age: 65-75	147	16	20	35	50	56	60	70	75	86	94	95
Male	Healthy	Age: 18-40	388	31	35	50	70	75	86	90	95	100	100	100
		Age: 41-64	396	40	50	70	80	86	90	95	100	100	100	100
		Age: 65-75	92	52	65	75	86	86	90	95	100	100	100	100
	At least one chronic condition	Age: 18-40	248	21	25	35	45	50	56	60	70	80	99	100
		Age: 41-64	436	16	20	35	45	50	60	65	73	86	100	100
		Age: 65-75	105	25	31	45	56	60	70	77	80	92	99	100
**Sex x Health status x Education**				**Low HRQoL**		***-1 SD***			***Md***			***+1 SD***		**High HRQoL**
Sex	Health status	Education	**N**	**2.5%**	**5%**	**16%**	**30%**	**40%**	**50%**	**60%**	**70%**	**85%**	**95%**	**97.25%**
Female	Healthy	education: low	171	45	50	60	75	80	80	86	90	100	100	100
		education: middle	341	31	45	65	75	80	86	90	95	100	100	100
		education: high	184	45	50	70	80	86	86	90	95	100	100	100
	At least one chronic condition	education: low	374	16	25	35	45	50	60	70	75	80	95	95
		education: middle	477	16	25	35	50	56	60	65	75	86	95	100
		education: high	187	25	31	44	50	56	60	70	75	86	100	100
Male	Healthy	education: low	202	35	45	51	75	80	86	90	95	100	100	100
		education: middle	394	31	40	65	75	86	90	95	100	100	100	100
		education: high	280	35	40	70	80	86	90	95	100	100	100	100
	At least one chronic condition	education: low	277	16	20	35	45	50	56	60	70	86	95	100
		education: middle	314	20	25	35	45	50	56	65	75	86	100	100
		education: high	198	25	30	35	50	56	60	65	75	90	100	100
		Total	3399	25	31	45	56	65	75	80	86	100	100	100

Note. HRQoL: health-related quality of life; 50% percentiles represent 50% of the distribution corresponding to the median (Md); SD: standard deviation; values from −1 standard deviation (16%) to +1 standard deviation (85%) are within the normal range (i.e., not impaired HRQoL); values below 16% indicate impaired HRQoL and values above 85% indicate outstanding HRQoL.
